# Hyperglycemia-independent neonatal streptozotocin-induced retinopathy (NSIR) in rats

**DOI:** 10.3389/fphar.2024.1395887

**Published:** 2024-07-23

**Authors:** Yu Lin, Wenyu Du, Xiangyu Fu, Ling Huang, Yiwen Hong, Haishan Tan, Lirong Xiao, Xiang Ren, Yujiao Wang, Danian Chen

**Affiliations:** ^1^ Department of Ophthalmology, West China Hospital, Sichuan University, Chengdu, China; ^2^ Research Laboratory of Ophthalmology and Vision Sciences, State Key Laboratory of Biotherapy, West China Hospital, Sichuan University, Chengdu, China

**Keywords:** streptozotocin (STZ), neonatal STZ-induced retinopathy (NSIR), retinal progenitors, hyperglycemia, cell cycle arrest, DNA damage

## Abstract

**Introduction:** Chemicals, such as MNU (N-methyl-N-nitrosourea) and NaIO3 (sodium iodate), are widely used to induce retinal degeneration in rodents. Streptozotocin (STZ) is an analog of N-acetyl glucosamine in which an MNU moiety is linked to a hexose and has a special toxic effect on insulin-producing pancreatic β-cells. It is commonly used to induce hyperglycemia to model diabetes. While intracerebroventricular injection of STZ can produce Alzheimer's disease independent of hyperglycemia, most retinal studies using STZ focus on the effects of hyperglycemia on the retina, but whether STZ has any impact on retinal cells independent of hyperglycemia is unknown. We aimed to investigate the role of cytotoxicity of STZ in rat retina.

**Methods:** Intravitreal or subcutaneous injection of STZ was performed on newborn rats. Electroretinogram (ERG) and H&E staining investigated retinal function and morphological changes. Retinal cell types, cell death, proliferation, inflammation, and angiogenesis were studied by immunostaining. RNA sequencing was performed to examine the transcriptome changes of retinal cells after intravitreal injection of STZ.

**Results:** Intravitreal (5 μg or 10 μg) or subcutaneous (30 mg/kg) injection of STZ at the early stage of newborn rats couldn’t induce hyperglycemia but caused NSIR (Neonatal STZ-induced retinopathy), including reduced ERG amplitudes, retinal rosettes and apoptosis, cell cycle arrest, microglial activation, and delayed retinal angiogenesis. STZ did not affect the early-born retinal cell types but significantly reduced the late-born ones. Short-term and long-term hyperglycemia had no significant effects on the NSIR phenotypes. RNA sequencing revealed that STZ induces oxidative stress and activates the p53 pathway of retinal cells. Locally or systemically, STZ injection after P8 couldn’t induce SINR when all retinal progenitors exit the cell cycle.

**Conclusion:** NSIR in rats is independent of hyperglycemia but due to STZ’s direct cytotoxic effects on retinal progenitor cells. NSIR is a typical reaction to STZ-induced retinal oxidative stress and DNA damage. This significant finding suggests that NSIR may be a valuable model for studying retinal progenitor DNA damage-related diseases, potentially leading to new insights and treatments.

## 1 Introduction

Experimental animal models are the keystone of modern biomedical research. They have been used to enhance the understanding of the basic pathophysiological mechanisms that underlie many retinal diseases, including inherited retinal degeneration (RD), age-related macular degeneration (AMD), glaucoma, and diabetic retinopathy (DR) (Niwa et al., 2016). Common animal models of retinal diseases include genetic, gene knockout, and transgenic models, such as the *rd1* mouse model for RD and the *RbKO* mouse model for retinoblastoma (Chen et al., 2004; Maeda and Maeda, 2018). Mechanical and chemical-induced models are also widely used in the eye research field, for instance, microbeads injection into the anterior chamber and intravitreal injection of *N*-methyl-d-aspartate (NMDA) to model glaucoma (Kluge and Reinehr, 2024). In addition, animal models with drug-induced retinal degeneration, including sodium iodate (NaIO3) and N-methyl-N-nitrosourea (MNU), have been extensively used, which specifically induce cell death of retinal pigment epithelium (RPE) cells and photoreceptors, and mimic AMD and RD, respectively (Reisenhofer et al., 2017). These models have been invaluable in developing new drugs and treatment modalities for these disorders.

STZ is a cytotoxic analog of N-acetyl glucosamine, in which the MNU moiety is linked to the C-2 of a hexose and has a special toxic effect on insulin-producing pancreatic β-cells (Lenzen, 2008; Eleazu et al., 2013). STZ induces DNA damage and activates poly (ADP) ribose polymerase (PARP-1), leading to the depletion of NAD ^(+)^ and ATP pool, which finally results in cell death (Yamamoto et al., 1981; Saini et al., 1996; Burkart et al., 1999). STZ is widely used to model diabetes in neonatal and adult rodents (Grieb, 2016). After systemic delivery, STZ is rapidly metabolized in the liver and quickly eliminated by renal excretion (Karunanayake et al., 1974). The half-life of STZ is short, only about 15 min in the serum after injection (Eleazu et al., 2013).

The selective cytotoxicity of STZ against pancreatic β-cells is mediated by its cellular uptake by the glucose transporter 2 (Glut2) (Thorens et al., 1988; Grieb, 2016). Systemically administered STZ can also damage other organs expressing low levels of Glut2, such as the kidneys and liver (Schmezer et al., 1994). Indeed, a major concern for the STZ-induced mouse model of diabetic nephropathy is the nonspecific toxicity of STZ to the kidney (Breyer et al., 2005).

The brain is generally unaffected by systemically delivered STZ because the blood-brain barrier lacks a Glut2 transporter (Grieb, 2016). Still, STZ administration directly through intracerebroventricular injection can produce Alzheimer’s disease-like lesions (Kamat, 2015). Glut2 is also expressed in the rat retina, likely in retinal progenitor cells (RPCs) and Müller glia, as linear staining along the outer limiting membrane (OLM) (Watanabe et al., 1994; Watanabe et al., 1999). While it is believed that Glut2 is not expressed in adult rat retinal neurons, Glut2 is strongly expressed in mouse horizontal cells (Yang et al., 2022). OLM is part of the blood-retinal barrier (BRB) (Omri et al., 2010); thus, rat retinal cells can uptake STZ from circulation. The expression level of Glut2 in the rat retina is similar between P6 (postnatal day 6) and adult rats but is 22.6-fold lower than in adult rat pancreas (Kermorvant-Duchemin et al., 2013). However, neonatal rat beta-cells express much fewer Glut2 transporters in the plasma membrane than adult rat beta-cells, which explains the insensitivity to extracellular glucose in neonatal beta-cells (Navarro-Tableros et al., 2007). Thus, we do not know if neonatal pancreas beta-cells are more sensitive to STZ than retinal progenitor cells.

The neonatal STZ rat model is a common animal model of diabetes (Portha et al., 1989; Baig and Panchal, 2019) and is widely used to study diabetic nephropathy (Shinde and Goyal, 2003; Gandhi et al., 2013) and diabetic neuropathy (Ashokkumar et al., 2006; Barragán-Iglesias et al., 2018). Most studies using the STZ-induced retinopathy animal models, including adult and neonatal rodent models, focus on the effects of hyperglycemia on the retina (Olivares et al., 2017), but whether STZ has direct and hyperglycemia-independent effects on rodent retinal cells is unknown. This study investigated whether intravitreal (IVIT) or subcutaneously (SC) injected STZ can cause retinal anomalies in neonatal rats. Some preliminary results of this study have been presented in the ARVO 2022 abstract (Lin et al., 2022).

## 2 Materials and methods

### 2.1 Animals

Timed pregnant Sprague Dawley (SD) rats were purchased from Vital River Laboratory Animal Technology Co., Ltd. (Beijing, China). They were delivered to the animal facility at West China Hospital (Chengdu, China) at around 17 days of gestation to allow for acclimatization before parturition. Access to water and standard rat chow was given *ad libitum*, and animals were provided with a 12-h dark, 12-h light cycle. Rats were treated according to institutional and national guidelines. All animal procedures were reviewed and approved by the Ethical Review Committee of Animal Research of West China Hospital, Sichuan University, Chengdu, Sichuan Province, China (AUP# 2018008A), and performed in compliance with the ARVO statement for the use of animals in ophthalmic and visual research.

### 2.2 Intravitreal injection (IVIT) and subcutaneous (SC) injection of STZ

STZ (Sigma-Aldrich Chemical Co., Saint-Quentin Fallavier, France) was freshly prepared in citrate buffer (pH 4.5) (Solarbio, China) to a final concentration of 60 mg/mL. For intravitreal (IVIT) injection, freshly made STZ solution was diluted to 1 μg/μL, 5 μg/μL, and 10 μg/μL, respectively. One microliter (1 μL) STZ or citrate buffer was injected into the vitreous. Briefly, neonates were anesthetized on ice for 30–60 s, the eyelids were prized apart gently, and forceps were applied. A topical anesthetic (0.5% proparacaine hydrochloride) was applied dropwise to the eye before injection. Eyes were injected under a dissection microscope using a 30-gauge needle attached to a Hamilton syringe. A drop of ophthalmic antibacterial agent (Ofloxacin ophthalmic solution USP, 0.3%) was applied to the injected eyeball. For systemic delivery, 30 or 60 mg/kg body weight (0.5 or 1 μL/g body weight) was administered via subcutaneous (SC) injections in the abdomen. Control animals received an equal volume of citrate buffer (Buffer group). The pups were returned to their mothers after the injection. The animals remained with their mothers until 21 days of age. Animals were weighed, and tail blood was collected for glucose measurement using a standard glucometer (Contour™ Plus, Ascensia).

### 2.3 Histology, immunofluorescence, whole-mount retinal staining

For hematoxylin and eosin (H&E) staining, eyeballs were fixed for more than 24 h in 10% neutral buffered formalin, dehydrated subsequently in ethanol, embedded with paraffin, and cut into 4 μm sections. The sections were rehydrated in ethanol, stained with hematoxylin and eosin, and finally mounted.

For immunofluorescence, eyeballs were fixed for 45 min at 4 °C in 4% paraformaldehyde, embedded in OCT (TissueTek 4583), frozen at −80°C and cut into 12 μm sections on Superfrost slides. Antigen retrieval was performed as described in our previous report (Chen et al., 2007). Slides were incubated with blocking solution (1% donkey serum and 0.1% Triton X-100 in PBS) for 1 h, and then with primary antibodies including Ap2a (Santa Cruz, SC-8975); Chx10 (Bremner lab, University of Toronto); Cleaved caspase-3 (Cell Signaling 9661); Cone arrestin or ARR3 (Millipore, AB15282); γ-H2ax (Millipore, 05–636); Onecut2 or OC2 (R&D system AF6294); Ki67 (BD science Pharmingen 550609); PH3 (Upstate 05–598); Rhodopsin (Santa Cruz SC-57433) and Sox9 (Millipore AB5535), overnight at 4°C. Vascular endothelial cells were labeled by FITC-IB4 (Sigma, L2895). Primary antibodies or labeled cells were visualized using donkey anti-mouse, donkey anti-rabbit, and donkey anti-goat antibodies conjugated with Alexa-488, Alexa-568, or Alexa-647 (1:1000; Molecular Probes). Nuclei were counter-stained with DAPI (Sigma, D9542) and mounted with Mowiol medium.

Eyeballs were enucleated and incubated for 45 min for whole-mount staining with 4% paraformaldehyde in PBS. A circumferential incision was made around the limbus to harvest the retina. The retinas were incubated at 4°C with primary antibody Brn3 (Santa Cruz, SC-6026) and FITC-IB4 for 2 days, then with secondary antibody (donkey anti-goat Alexa Fluor 488) for a day at 4°C. After briefly washing with PBS, radial cuts were made to divide the retina into four quadrants to flatten the retina, and flat retinas were mounted with Mowiol.

Stained sections and slides were analyzed using a Zeiss Axio Imager Z2 fluorescence microscope and a Nikon C1si confocal microscope. The positive cells of cleaved caspase-3, Ki67, PH3, EdU, γ-H2ax, and cell-type markers (including Brn3, Rho, ARR3, Chx10, Ap2a, OC2, and Sox9) were counted manually or automatically with ImageJ 1.50b with a cell counter plugin (https://imagej.nih.gov/ij/). The minimal number of images for each group was 12 (two images per section, two sections per retina, and three retinas from three animals). Images of peripheral retinas (which have ciliary body and iris in the same image) and central retinas (which have optic nerve in the same image) were separately counted. Most images for cell counting were captured under a fluorescence microscope using a ×20objective lens. For Ki67 and Rho counting, 60 × objective lenses were also used. The quantitative variables of this study were presented as mean ± standard deviation (Mean ± SD) or standard error (SE). The thickness of ONL (outer nuclear layer) was measured using a microscope program. Representative images were analyzed using the AngioTool software (National Cancer Institute, United States) to quantify the average vessel length and branching points for vascular blood vessel analysis.

### 2.4 EdU labeling

To label the S phase of retinal progenitor cells, animals were injected subcutaneously once with EdU (Sigma, 900584, 30 μg/g of body weight) to label all cells in the S phase. The retinas were harvested 0.5 h after EdU injection, fixed in 4% paraformaldehyde for 1 h, and dehydrated in 30% sucrose for 24 h. For retinal sections, EdU was detected with the Click-iT^®^ EdU Alexa Fluor^®^ 647 Imaging Kit (Thermo Fisher, C10340) according to the manufacturer’s protocol.

### 2.5 RNA-sequencing

Total RNAs were extracted from dissected rat retinas using TRIzol (Thermo Fisher) and treated with RNAse-free DNAse I (New England Biolabs) to remove genomic DNA. The yield of total RNA was assessed using NanoDrop Microvolume Spectrophotometers (NanoDrop Technologies). The cDNA libraries were prepared using an Illumina TruSeq RNA sample preparation kit, and the quality was assessed using an Agilent 2100 Bioanalyzer (Agilent Technologies). For sequencing, the cDNA libraries were loaded on an Illumina HiSeq 2500 at Biomaker (Beijing, China). The raw sequence reads in FASTQ format were processed and analyzed as previously reported (Liu et al., 2014; Gregorieff et al., 2015). Briefly, the sequencing quality was first assessed using FastQC, and poor-quality 5′end reads were trimmed using a Perl script and then mapped onto the rat genome using TopHat2, allowing for up to 2 mismatches as default settings. Reads mapped onto multiple genomic locations were discarded, and a custom R script was used to calculate the fragments per kilobase million (FPKM) of each gene and obtain the expression profile of each sample (Supplementary Table S1). The expression folds change of each coding gene of the retinas of the STZ group compared with the retinas of the buffer group was calculated using the following formula: Fold change = (FPKM ^STZ^ +1)/(FPKM ^buffer^ +1). The genes for expression fold changes greater than 1.54 or less than 0.65 were selected as deregulated genes (DEGs). The heatmap was generated using Heatmapper (Babicki et al., 2016). The function enrichment of DEGs was performed using Enrichr (Chen et al., 2013; Kuleshov et al., 2016); the pathways with a *p*-value less than 0.05 were chosen to report.

### 2.6 Quantitative real-time PCR

Total RNA was isolated from rat retinas using TRIzol reagent (Thermo Fisher). After quantification by NanoDrop, first-strand cDNA was synthesized from 1 µg of total RNA using the RT reagent Kit with gDNA wiper (Vazyme, China). PCR primers are listed in Supplementary Table S2. Real-time quantitative PCR was performed using the qTOWER 2.2 PCR machine (Analytik Jena, Germany). Tests were run in duplicate on three separate biological samples with HiScript II Q RT SuperMix (Vazyme, China). PCR consisted of 40 cycles of denaturation at 95°C for 15 s, annealing, and extension at 55°C for 30 s. An additional cycle (95°C, 15 s) generated a dissociation curve to confirm a single product. Values obtained for test RNAs were normalized to β-actin mRNA levels.

### 2.7 Flow cytometry

According to the manufacturer’s instructions, single-cell suspension was prepared from the dissected rat retina with a dissociation kit (Papain Dissociation System Kit, Worthington). Then, the retinal cells were fixed in 70% cold ethanol and incubated with propidium iodide solution (Sungene Biotech, China) before running them on the flow cytometer (FACSAria™ III Cell Sorter, BD). Data were analyzed by ModFit LT 5.0 (Verity Software House, Topsham, ME, United States).

### 2.8 Electroretinography (ERG)

Dark-adapted ERG (RetMINER™, IRC, Chongqing, China) were recorded at P21, P28, P42, and P180. The procedures were performed as previously described (Zheng et al., 2018). In brief, rats were dark-adapted overnight and anesthetized by IP injection of ketamine (100 mg/kg) and xylazine (15 mg/kg). Pupils were dilated with 0.5% tropicamide and 0.5% phenylephrine hydrochloride. A full-field ERG was recorded after subcutaneously inserting a ground electrode near the tail and a reference electrode on the head. A golden-ring electrode was gently positioned on the cornea. All procedures were performed under dim red light. We used GraphPad to illustrate and analyze the data recorded by the ERG device. Because there was no light stimulation in the first 25 s, we used the baseline correction function in GraphPad to normalize the baseline to around 0 μV. Before we measured the amplitude of a-wave and b-wave, we used the bandpass filter function in Clampfit 11.1 software (Molecular Devices) to filter clutter and only used waves with a frequency between 0.3 Hz and 65 Hz in the final version of the dark-adapted ERG. The amplitude of the a-wave was measured from the baseline to the peak of the a-wave, and the b-wave was measured from the trough of the a-wave to the apex of the b-wave. Oscillatory potentials (OPs) were studied using Clampfit 11.1 software (Molecular Devices). The extracted OPs were analyzed by Fast Fourier Transform (FFT) and converted to a single-sided smoothed frequency power spectrum with functions built in the Clampfit. To evaluate the OPs magnitude, areas enclosed by the frequency spectrum curve were calculated to assess the total power of OPs using GraphPad.

### 2.9 Statistical analysis

All data were presented as mean ± SD or SE. Statistical analysis was undertaken using the GraphPad Prism software (GraphPad Prism Software, Inc., San Diego, CA, United States). The results were analyzed by one-way analysis of variance (ANOVA) followed by Bonferroni correction for multiple comparisons. The threshold for significance was set at *p* < 0.05.

## 3 Results

### 3.1 Intravitreal injection of STZ can induce NSIR without hyperglycemia

**FIGURE 1:**
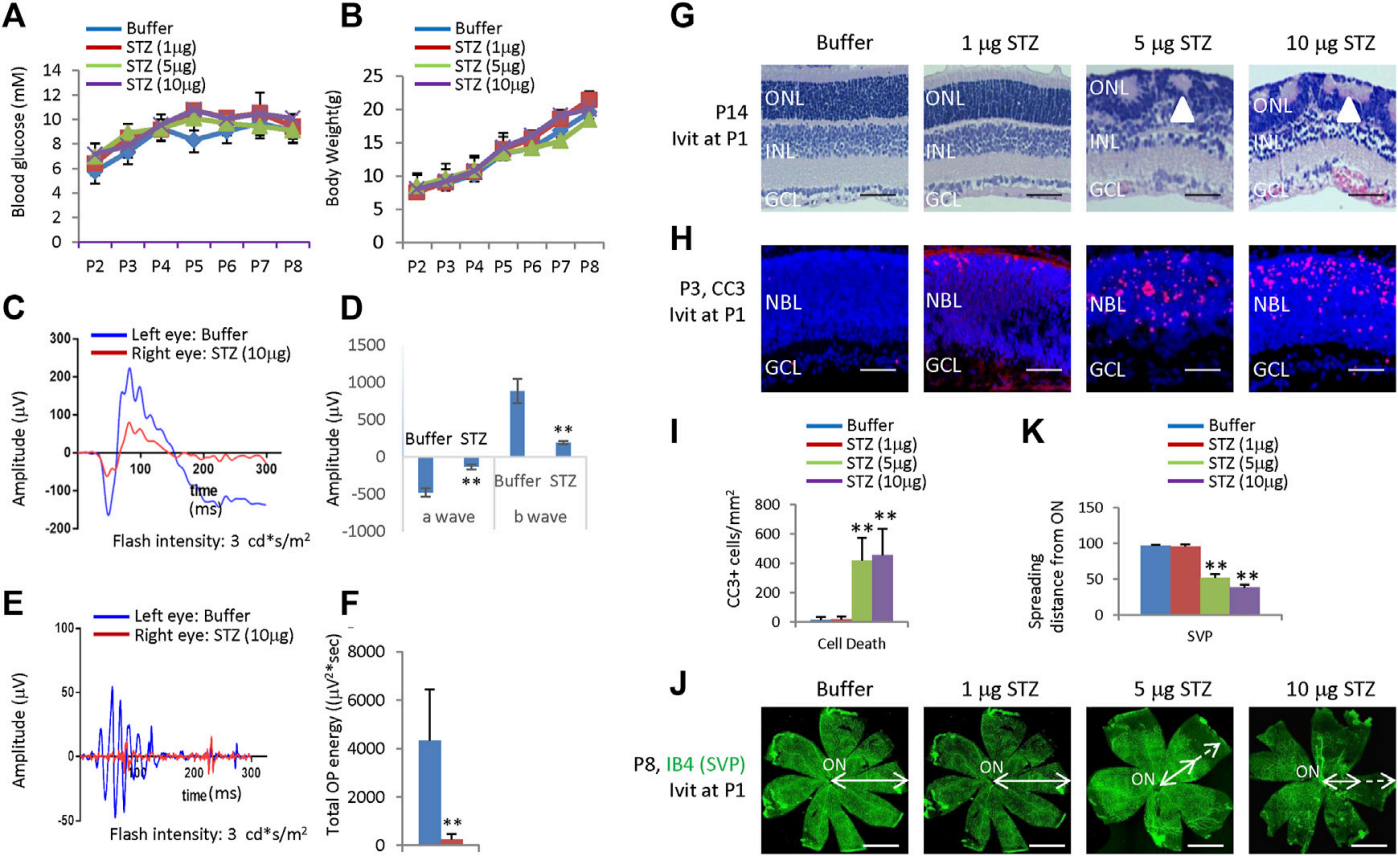
Blood glucose, body weight, ERG, retinal morphological changes, retinal apoptosis, and retinal angiogenesis in intravitreal STZ-treated rats at P1. **(A)** Blood glucose level from P2 to P8 of indicated groups receiving an intravitreal injection at P1. **(B)** Body weight from P2 to P8 of indicated groups receiving an intravitreal injection at P1. **(C)** ERG responses at P21 to flashes of the intensity of 3.0 cd*s/m^2^. **(D)** Amplitude of a wave and b wave in **(C)**. **(E)** The OPs were extracted from dark-adapted ERGs at the intensity of 3.0 cd*s/m^2^ at P21. **(F)** Total energy of the extracted OPs in **(E) (G)** H&E staining of P14 retinas of the buffer and STZ-treated rats. White arrowheads indicate retinal rosettes. **(H)** Cleaved caspase 3 staining of P3 retinas in the control and STZ-treated rats. **(I)** Quantification of Cleaved caspase 3+ cells per mm^2^ area of the retina. **(J)** IB4 staining of P8 whole-mount retinas of indicated groups. Bidirectional arrows indicate the spreading distance of vascular vessels from the optic nerve (ON). Dashed arrows indicate areas without blood vessels. **(K)** Quantifying the spreading distance of vascular vessels from ON in **(D)** indicated groups. Error bars represent SD of measurements from three animals or three retinas of three animals (n = 3), and asterisks indicate significant differences between control and STZ-treated groups (*p < 0.05, **p < 0.01, one-way ANOVA followed by Bonferroni’s correction). ONL, outer nuclear layer; INL, inner nuclear layer; GCL, ganglion cell layer; NBL, neuroblast layer; SVP, retinal superficial vessel plexus; ON, optic nerve. Scale bar: 50 μm **(G,H)**, or 500 μm **(J)**.

Intravitreal injection (IVIT) of STZ 1 μg, 5 μg, and 10 μg at postnatal day 1 (P1) did not affect glucose and weight (Figures 1A, B). To test if STZ has direct cytotoxicity to neonatal rat retina, we IVIT 1 μg, 5 μg, and 10 μg STZ at P1 to the right eyes. At the same time, an equal amount of citrate buffer was injected into the left eye of the same animal. We then examined the function and morphology of the retina.

**FIGURE 2:**
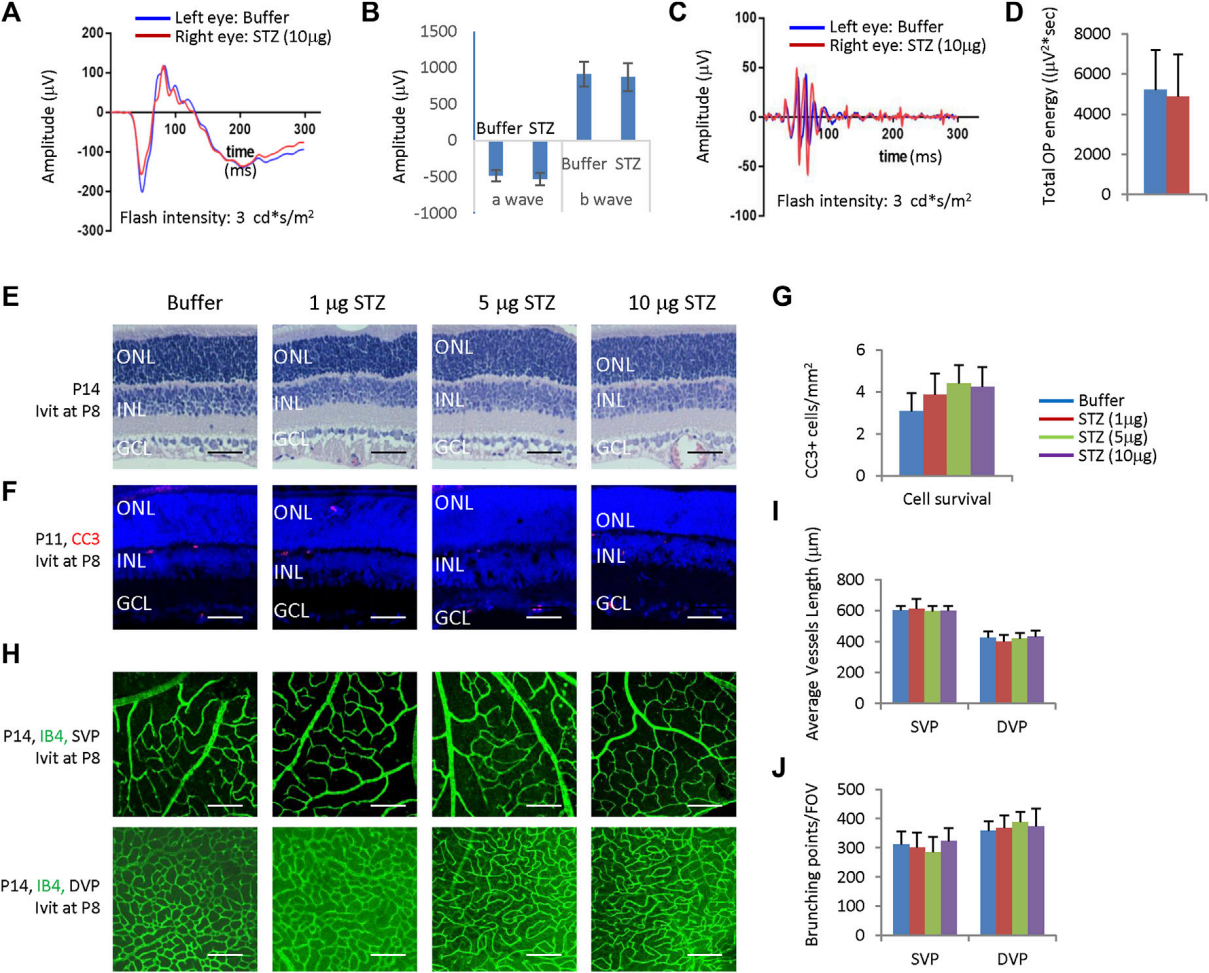
Intravitreal STZ injection at P8 does not affect ERG, retinal morphology, retinal apoptosis, and angiogenesis. **(A)** ERG responses at P28 to flashes of the intensity of 3.0 cd*s/m^2^. **(B)** The amplitude of a wave and b wave in **(A) (C)** The OPs extracted from dark-adapted ERGs at 3.0 cd*s/m^2^ at P28. **(D)** Total energy of the extracted OPs in **(C)**. **(E)** H&E staining of P14 retinas of the buffer and STZ-treated rats. **(F)** Cleaved caspase 3 staining of P11 retinas of the buffer and STZ-treated rats. **(G)** Quantification of Cleaved caspase 3+ cells per mm^2^ area of the retina. **(H)** IB4 staining of P14 whole-mount retinas of indicated groups. **(I)** Quantification of the average vessel length of SVP/DVP of indicated groups. **(J)** Quantifying the branching points of SVP/DVP indicated groups. Error bars represent the SD of measurements from three animals or three retinas of three animals (n = 3), and asterisks indicate significant differences between control and STZ-treated groups (*p < 0.05, **p < 0.01, one-way ANOVA followed by Bonferroni’s correction). ONL, outer nuclear layer; INL, inner nuclear layer; GCL, ganglion cell layer; SVP, retinal superficial vessel plexus; DVP, retinal deep vessel plexus; FOV, field of view; Ivit, Intravitreal injection. Scale bar: 50 μm **(E,F)**, or 200 μm **(H)**.

ERG measured at P21 showed that intravitreal STZ (10 μg) reduced the amplitudes of dark-adapted a-waves and b-waves (Figures 1C, D), and the total OPs energy at the light stimulation intensity of 3.0 cd*s/m^2^ (Figures 1E,F). H&E staining of P14 retinal sections identified ONL rosettes in the intravitreal STZ (5–10 μg) injected group (Figure 1G), but 1 μg intravitreal STZ has no effects on retinal morphology (Figure 1G). Cleaved caspase 3 (CC3) staining found that IVIT 5–10 μg STZ induced widespread retinal apoptosis at P3, but 1 μg STZ had only induced a few apoptotic retinal cells (Figures 1H, I). IB4 staining at P8 showed that 5–10 μg intravitreal STZ treatments delayed the retinal angiogenesis, as retinal SVP had not reached the peripheral in both groups, but 1 μg STZ had no measurable effects on retinal angiogenesis (Figures 1J, K). Intravitreal injection of 5–10 μg STZ at P8 did not affect retinal function (Figures 2A–D) and morphology (Figure 2E), survival (Figures 2F, G), and angiogenesis (Figures 2H–J). These results suggested that STZ can directly induce neonatal retinal abnormalities independent of hyperglycemia, but only when STZ was injected at an early stage of retinal development (P1).

### 3.2 STZ suppresses RPCs proliferation and delays their cell cycle exit

**FIGURE 3:**
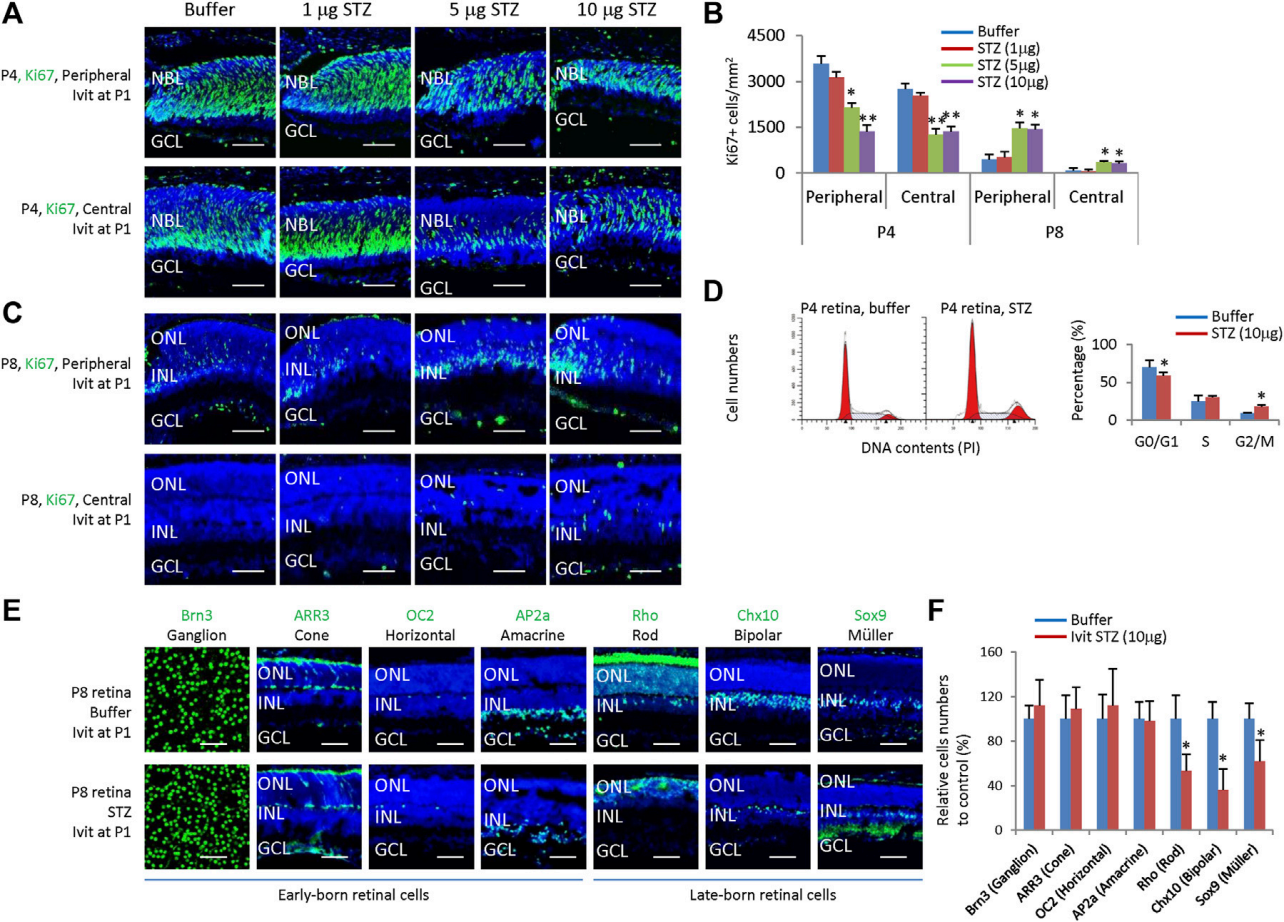
Intravitreal STZ injection on P1 inhibits the proliferation of neonatal rat retinal progenitors and delays their cell cycle exit and differentiation. **(A)** Horizontal P4 retinal sections of indicated locations and groups were stained for nuclear (DAPI, blue) and cell proliferation (Ki67, green). **(B)** Quantification of Ki67+ cells per mm^2^ area of P4 and P8 retinas. **(C)** Horizontal P8 retinal sections of indicated locations and groups were stained for nuclear (DAPI, blue) and cell proliferation (Ki67, green). **(D)** Flow cytometry analysis of P4 retinal cells. **(E)** Whole-mount or horizontal sections of P8 retinas of indicated groups were stained for nuclear (DAPI, blue) and cell type markers, including Ganglion cells (Brn3, green, whole-mount), Cone (ARR3, green), Horizontal cells (OC2, green), Amacrine cells (Ap2a, green), Rod (Rho, green), Bipolar cells (Chx10, green) and Müller glia (Sox9, green). **(F)** Quantifying all seven retinal cell types (relative to the control group, %). Error bars represent SD of measurements from three animals or three retinas of three animals (n = 3), and asterisks indicate significant differences between control and STZ-treated groups (*p < 0.05, **p < 0.01, one-way ANOVA followed by Bonferroni’s correction). ONL, outer nuclear layer; INL, inner nuclear layer; GCL, ganglion cell layer; Ivit, Intravitreal injection; PI, Propidium iodide. Scale bar: 50 μm.

STZ only affects neonatal rat retinal cells in the early stage (P1); locally administered STZ after P8 had no effects (Figure 2). Thus, it is very likely that STZ mainly affects retinal progenitor cells (RPCs). Indeed, IVIT 5–10 μg STZ at P1 significantly reduced the number of ki67+ cells at P4 in the central and peripheral retina (Figures 3A, B). However, at P8, while most RPCs exit the cell cycle, IVIT 5–10 μg STZ at P1 significantly increased the number of ki67+ cells (RPCs) in the central and peripheral retina (Figures 3B, C). Flow cytometry analysis of P4 retinal cells showed that STZ reduced the G0/G1 phase but increased the G2/M phase while having no effects on the S phase (Figure 3D). PH3 staining indicated that STZ significantly reduced the M phase (PH3+) cells (Supplementary Figure S1). Thus, STZ blocked the cell cycle of RPCs at the G2 phase. This result suggested that STZ might increase the cell cycle length of RPCs and delay their cell cycle exit and differentiation.

Consistent with this notion, IVIT STZ (10 µg) at P1 had no effects on the numbers of early-born retinal cells, including ganglion cells (Brn3+), horizontal cells (OC2+), amacrine cells (AP2a+) and cones (ARR3+) (Figures 3E, F), but significantly reduced late-born retinal cells, including rods (Rho+), bipolar cells (Chx10+) and Müller glia (Sox9+) (Figures 3E, F). Thus, we concluded that STZ mainly affects the survival and proliferation of RPCs in neonatal rats.

### 3.3 STZ induces DNA damage and oxidative stress, activates the p53 and inflammation pathway

Based on the above results, locally administered STZ at P1 could induce retinal cell death, suppress RPCs proliferation, and delay retinal differentiation and angiogenesis. To understand the underlying mechanism, we analyzed the transcriptome of the P4 retinas of rats receiving IVIT STZ (10 g) by RNA sequencing.

**FIGURE 4:**
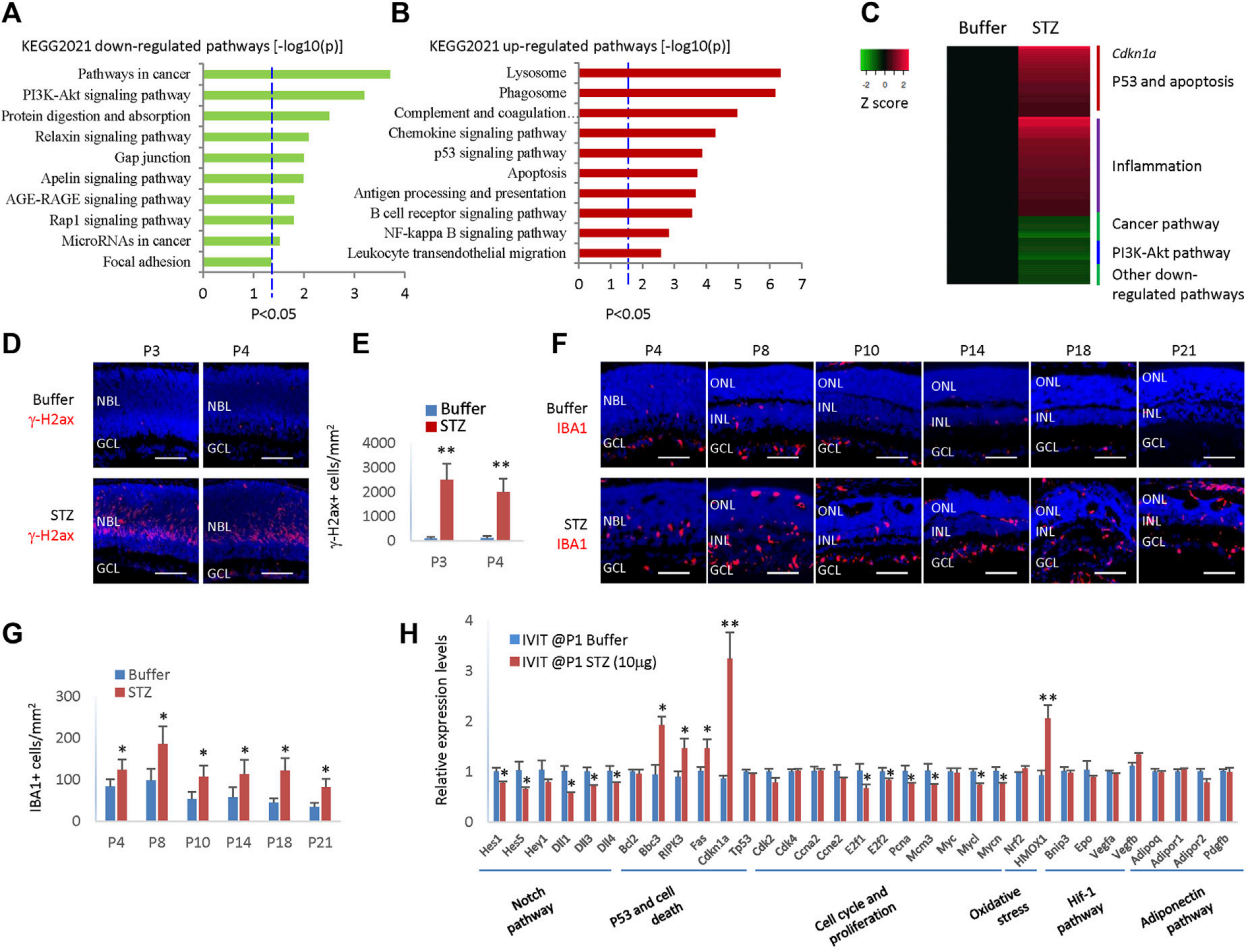
STZ induces cell death and inflammation pathways and p21cip1 in neonatal rat retina. **(A)** Gene list enrichment analysis using KEGG 2021 datasets in Enrichr of STZ downregulated DEGs (−log10(P)). Dotted line, *p* < 0.05. **(B)** Gene list enrichment analysis using KEGG 2021 datasets in Enrichr of STZ upregulated DEGs (−log10(P)). Dotted line, *p* < 0.05. **(C)** Heatmap of top de-regulated pathways of STZ-regulated DEGs by RNA sequencing. **(D)** P3/P4 horizontal retinal sections of indicated groups were stained for nuclear (DAPI, blue) and DNA damage (γ-H2ax, red). **(E)** Quantifying retinal cells with DNA damage (γ-H2ax +) per mm^2^ area of the retina. **(F)** Horizontal retinal sections of indicated time points and groups were stained for nuclear (DAPI, blue) and microglia (IBA1, red). **(G)** Microglial cells (IBA1+) are quantified per mm^2^ area of the retina. **(H)** RT-PCR analyzed the relative mRNA levels of indicated genes and indicated groups. Error bars represent SD **(E,G)** or SE **(H)** of measurements from three animals or three retinas of three animals (n = 3), and asterisks indicate significant differences between control and STZ-treated groups (*p < 0.05, **p < 0.01, one-way ANOVA followed by Bonferroni’s correction). NBL is the neuroblast layer; ONL is the outer nuclear layer; INL is the inner nuclear layer; GCL is the ganglion cell layer. Scale bar: 50 μm.

We had two control groups: citrate buffer and saline. We found no enrichment of deregulated genes (DEGs) between citrate buffer and saline. Thus, we focused on the differences between the STZ and citrate buffer groups. We identified 608 STZ-related DEGs, including 416 upregulated DEGs and 192 downregulated DEGs (Supplementary Table S1). Gene list enrichment analysis by Enrichr indicated that the most downregulated STZ-related DEGs were in the cancer pathway and PI3K-Akt pathway (Figure 4A), corresponding to RPCs cell cycle arrest and delayed retinal angiogenesis. The most enriched upregulated pathways were related to cell death and inflammation (Figures 4B, C). The p53 pathway, apoptosis pathway, phagosome, and lysosome pathways may be linked to STZ-induced RPCs death (Figures 4B, C).

It is well known that STZ can induce DNA damage via DNA alkylation. DNA damage can activate the p53 pathways, thus activating many genes whose products trigger cell-cycle arrest, apoptosis, or DNA repair. Indeed, many γ-H2ax + cells were in the STZ-treated retinas at P3 and P4 (Figures 4D, E). The expression of Cdkn1a increased four times in the STZ-treated retina (Figure 4C), which may be a major mechanism of cell cycle arrest as Cdkn1a is a well-known cell cycle inhibitor.

Many inflammation pathways were activated, including the complement, chemokine pathway, NF-kB pathway, and phagocytosis pathway (Figure 4C). Thus, likely STZ-induced RPCs death initiated a profound inflammation reaction in the neonatal rat retinas. Indeed, the IBA1+ microglia increased from P4-P21 in STZ-treated retinas (Figures 4F, G). Consistent with the RNA sequencing result, RT-PCR confirmed that p53 pathway genes (*Bbc3, Ripk3, Fas,* and *cdkn1a*) were significantly upregulated. In contrast, cell cycle genes (*E2f1, E2f2, Pcna, Mcm3, Mycl, and Mycn*) were significantly downregulated in STZ-treated retinas (Figure 4H).

In addition, the Notch pathway (for instance, genes of *Hes1*, *Hes5*, *Hey1*, *Dll1*, *Dll3,* and *Dll4*) were also significantly downregulated, explaining the delayed retinal differentiation (Figure 4H). STZ also induced retinal oxidative stress, as the *heme oxygenase-1* (*Hmox-1*) gene was hugely induced (Figure 4H). STZ had no effects on Hif-1 pathway and adiponectin pathway genes (Figure 4H).

### 3.4 Systemic-delivered 30 mg/kg STZ introduced NSIR without hyperglycemia

**FIGURE 5:**
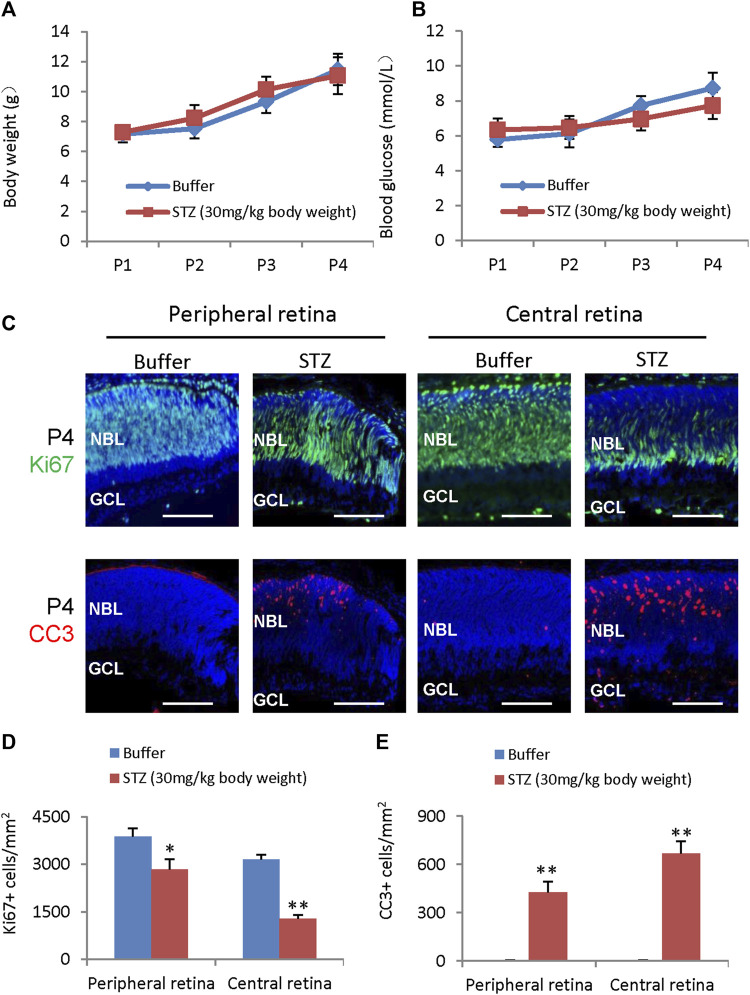
STZ-induced neonatal retinopathy is independent of hyperglycemia. STZ (30 mg/kg body weight) was injected at P1 and P3. **(A)** The body weight of control and STZ groups. **(B)** The blood glucose levels of control and STZ groups. **(C)** Horizontal P4 retinal sections were stained for nuclear (DAPI, blue), cell division (Ki67, green), and apoptosis (cleaved caspase 3, or CC3, red). **(D)** Quantification of Ki67+ cells per mm^2^ area of peripheral and central retinas. **(E)** Quantification of cleaved caspase 3 (CC3)+ cells per mm^2^ area of the retina. Error bars represent SD of measurements from three animals or three retinas of three animals (n = 3), and asterisks indicate significant differences between control and STZ-treated groups (*p < 0.05, **p < 0.01, one-way ANOVA followed by Bonferroni’s correction). NBL is the neuroblast layer; GCL is the ganglion cell layer. Scale bar: 50 μm.

Even though locally injected STZ can induce NSIR, we do not know if systemically delivered STZ can induce similar retinal lesions, as BRB (blood-retinal barrier) may block transferring STZ from circulation into the retina. STZ (30 mg/kg of body weight) was subcutaneous (SC) injected at P1 and P3, and rats were analyzed at P4. The weight (Figure 5A) and blood glucose (Figure 5B) were the same between the control and STZ groups. However, STZ suppressed the proliferation of RPCs (Figures 5C, D) and induced retinal apoptosis (Figures 5C, E). Thus, systemically delivered STZ (low dosage) could pass the BRB and induce NSIR without causing hyperglycemia. This suggests that retinal progenitors may be more sensitive to STZ than neonatal pancreatic β-cells.

### 3.5 Hyperglycemia has no significant effects on NSIR

While locally or systemically injected STZ can induce NSIR without causing hyperglycemia, we do not know if STZ-induced hyperglycemia has a role in the pathogenesis of NSIR. To directly assess the impact of hyperglycemia on the phenotypes of NSIR, we increased the dosage of STZ from 30 mg/kg to 60 mg/kg for SC injection and injected STZ at different time points. For the SC STZ 60 mg/kg body weight cohort, we did not observe any rat pup death in the P1/P3/P5 and P2/P4/P6 injection group, but in the P3/P5/P7, P4/P6/P8 and P8/P10 injection groups, we found that about half of pups receiving this dosage of STZ died in 1–2 days after the second injection at P6-P12, likely due to hyperglycemia.

#### 3.5.1 Inducing hyperglycemia by SC injection of STZ

**FIGURE 6:**
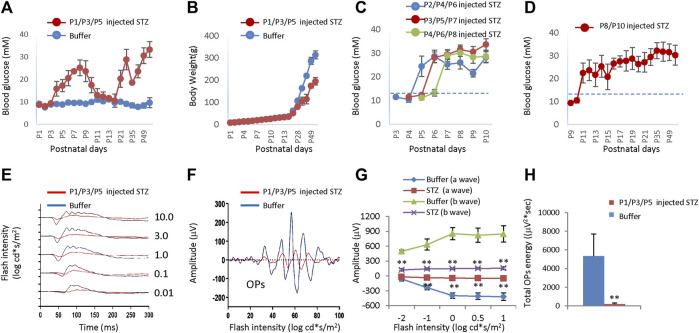
Blood glucose level, body weight, and ERG of neonatal rats after subcutaneous injection of STZ or buffer at different time points. **(A)** The blood glucose levels of the two groups were injected at P1/P3/P5. **(B)** Body weight of two groups injected at P1/P3/P5. **(C)** Blood glucose levels of rats got STZ injection at P2/P4/P6, P3/P5/P7, and P4/P6/P8, respectively. **(D)** Blood glucose level of rats got STZ injection at P8/P10. **(E)** Scotopic ERG responses to flashes of increasing intensities from 0.01 to 10.0 cd*s/m2 at P21. **(F)** The OPs were extracted from dark-adapted ERGs at the intensity of 3.0 cd*s/m2 at P21. **(G)** Amplitude of a wave and b wave. **(H)** The total energy of the extracted OPs (Total energy, an alternative measurement of OPs amplitude, is qualified by calculating the area enclosed by the frequency power spectrum). Dash lines in C/D indicated normal blood glucose levels (13.3 mM). Error bars represent SD of measurements from six animals (n = 6), and asterisks indicate significant differences between control and STZ-treated groups (*p < 0.05, **p < 0.01, one-way ANOVA followed by Bonferroni’s correction). In Fig. E–H, STZ was injected at P1/P3/P5, and ERG was measured at P21.

SC injection of STZ (60 mg/kg body weight) at P1, P3, and P5 (P1/P3/P5) led to consistent hyperglycemia. Hyperglycemia was observed after the second injection with a mean blood glucose of 18 ± 1.14 mmol/L at P4 (Figure 6A). Hyperglycemia was sustained for a week until P9 when the mean glucose was 22.1 ± 0.99 mmol/L (Figure 6A). In the second week after birth, the blood glucose returned to normal, then increased again after P21 (20.3 ± 3.8 mmol/L), lasting at least 2 months (Figure 6A). Weight gain was not affected in STZ-injected rats in the first 2 weeks (Figure 6B). Nevertheless, starting from the third week, the hyperglycemic rats gained much less weight (Figure 6B). We also injected STZ at different time points. SC injection of 60 mg/kg STZ at P2/P4/P6, P3/P5/P7, P4/P6/P8 and P8/P10 got similar hyperglycemia without any period of normoglycemia (Figures 6C, D), indicating no recovery of β cell mass when injection of STZ at these time points.

#### 3.5.2 Retinal function and structure changes after SC injection of 60 mg/kg STZ at P1/P3/P5

**FIGURE 7:**
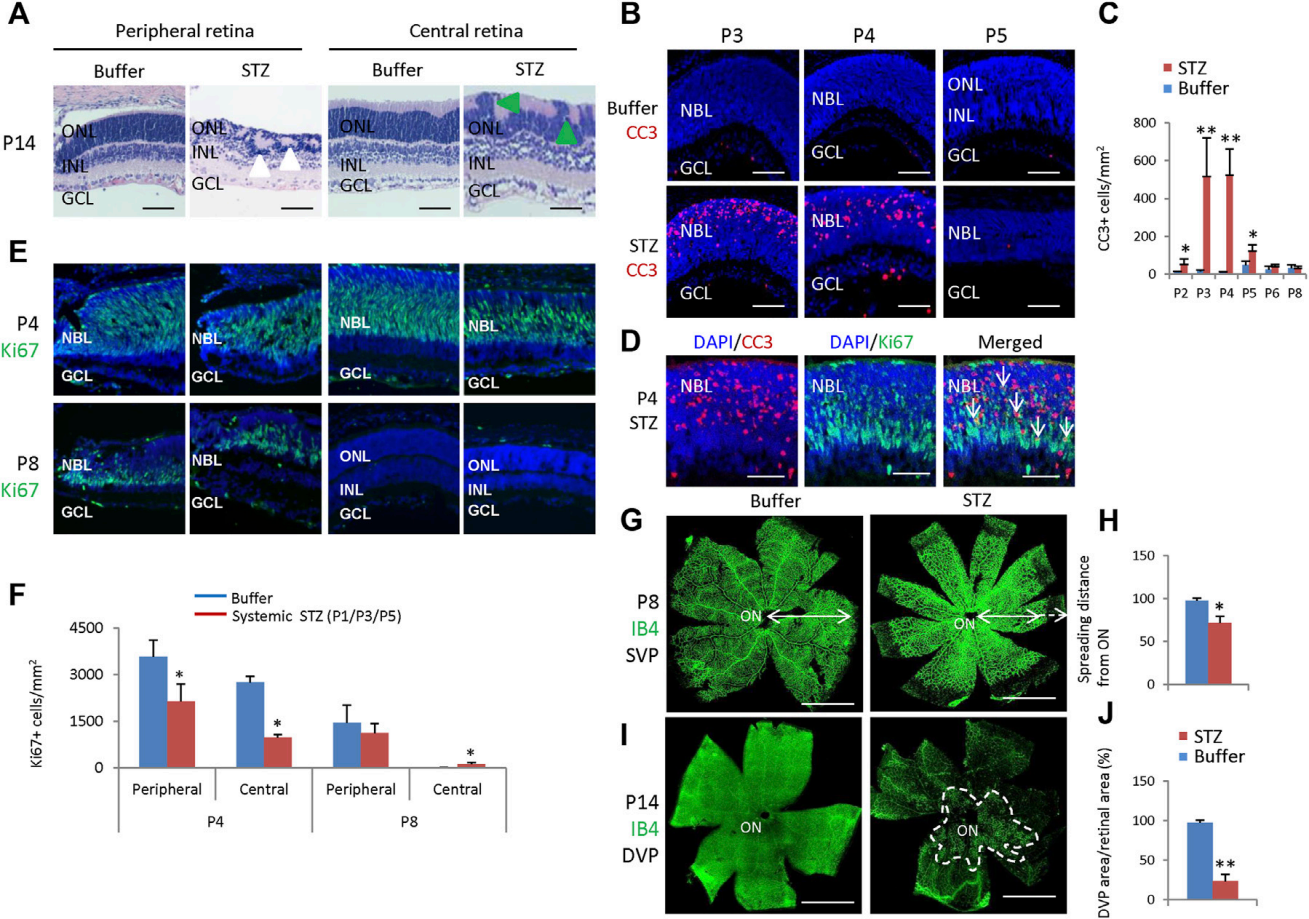
Retinal morphological changes, apoptosis, and angiogenesis in STZ-treated rats (P1/P3/P5). **(A)** H&E staining of P14 retinas of the control and STZ-treated rats. White arrowheads indicate retinal rosettes and green arrowheads indicate retinal dysplasia. **(B)** Cleaved caspase 3 staining of P3-P5 retinas of the control and STZ-treated rats. **(C)** Quantification of Cleaved caspase 3 (CC3)+ cells per mm^2^ area of the retina. **(D)** Cleaved caspase 3 (red) and Ki67 (green) staining of P4 STZ-treated retinas. Arrows indicate dying, dividing retinal progenitor cells. **(E)** Horizontal P4 and P8 retinal sections of indicated locations and groups were stained for nuclear (DAPI, blue) and cell proliferation (Ki67, green). **(F)** Quantification of Ki67+ cells per mm^2^ area of P4 and P8 retinas. **(G)** IB4 staining of P8 whole-mount retinas of indicated groups. Bidirectional arrows indicate the spreading distance of vascular vessels from the optic nerve (ON). Dashed arrows indicate areas without blood vessels. **(H)** Quantifying the spreading distance of vascular vessels from ON in D of indicated groups. **(I)** IB4 staining of the DVP of P14 whole-mount retinas of indicated groups. The dashed white line outlined the location of DVP in the STZ-treated retina. **(J)** Quantification of the DVP areas per whole retinal area (%). Error bars represent SD of measurements from six retinas of six animals (n = 6), and asterisks indicate significant differences between control and STZ-treated groups (*p < 0.05, **p < 0.01, one-way ANOVA followed by Bonferroni’s correction). ONL, outer nuclear layer; INL, inner nuclear layer; GCL, ganglion cell layer; NBL, neuroblast layer; SVP, retinal superficial vessel plexus; DVP, retinal deep vessel plexus; ON, optic nerve. Scale bar: 50 μm **(A,B,D,E)**, or 500 μm **(G,I)**.

After three SC injections of 60 mg/kg STZ on P1/P3/P5, we examined the function and morphology of the retina. ERG indicated that STZ-treated rats had abnormal scotopic a-wave, b-wave, and OPs at P21 (Figures 6E, F). The amplitudes of dark-adapted a-wave and b-waves were all significantly decreased over a wide range of intensities from 0.01 to 10.0 cd*s/m^2^ (Figure 6G). The total OPs energy at the light stimulation intensity of 3.0 cd*s/m^2^ was also reduced in the STZ-injected group (Figure 6H). Consistent with ERG defects, H&E staining of P14 retinal sections identified retinal morphological changes in the STZ-injected group (Figure 7A). These changes included peripheral retinal rosettes and central retinal dysplasia (Figure 7A). These retinal functional changes and morphological lesions were similar to IVIT STZ-induced NSIR (Figures 1C–G).

#### 3.5.3 Retinal cell death, proliferation, and angiogenesis after SC injection of 60 mg/kg STZ at P1/P3/P5

CC3 staining found that STZ induced widespread retinal apoptosis, with the peak at P3-P4 (Figures 7B,C). TUNEL labeling also found similar results (Supplementary Figure S2). These cell deaths were also similar to IVIT STZ-induced NSIR (Figures 1H, I). Many CC3+ cells were also Ki67+, indicating they were dying retinal progenitor cells (RPCs) (Figure 7D). However, there were also many Ki67-negative dying cells; the Ki67 protein was likely degraded in these cells (Figure 7D).

SC 60 mg/kg injection of STZ significantly reduced the number of ki67+ cells at P4, at both the central and peripheral retina (Figures 7E, F). However, at P8, most RPCs exit the cell cycle; systemic STZ increased the number of ki67+ RPCs in both the central retinas (Figures 7E, F). Similar results were also observed in the EdU labeling assay (Supplementary Figure S3). Systemic STZ might increase the cell cycle length of RPCs and delay their cell cycle exit, as illustrated by EdU/Ki67 labeling in P6 (Supplementary Figure S4) and P8 (Supplementary Figure S5) retinas. These effects on the cell cycle of retinal progenitor cells were similar to IVIT STZ-induced NSIR (Figures 3A–D).

STZ also delayed retinal angiogenesis. At P8, the retinal superficial vessel plexus (SVP) of STZ-treated rats had not reached the peripheral (Figures 7G, H). At P14, only a tiny portion of the retinal deep vessel plexus (DVP) had developed (Figures 7I, J).

Thus, systemic injection of 60 mg/kg STZ to rats on P1/P3/P5 can induce hyperglycemia and NSIR. The latter was similar to IVIT STZ-induced NSIR, suggesting hyperglycemia had no major effects on NSIR.

#### 3.5.4 Retinal changes in rats receiving SC injection of STZ at other time points

**FIGURE 8:**
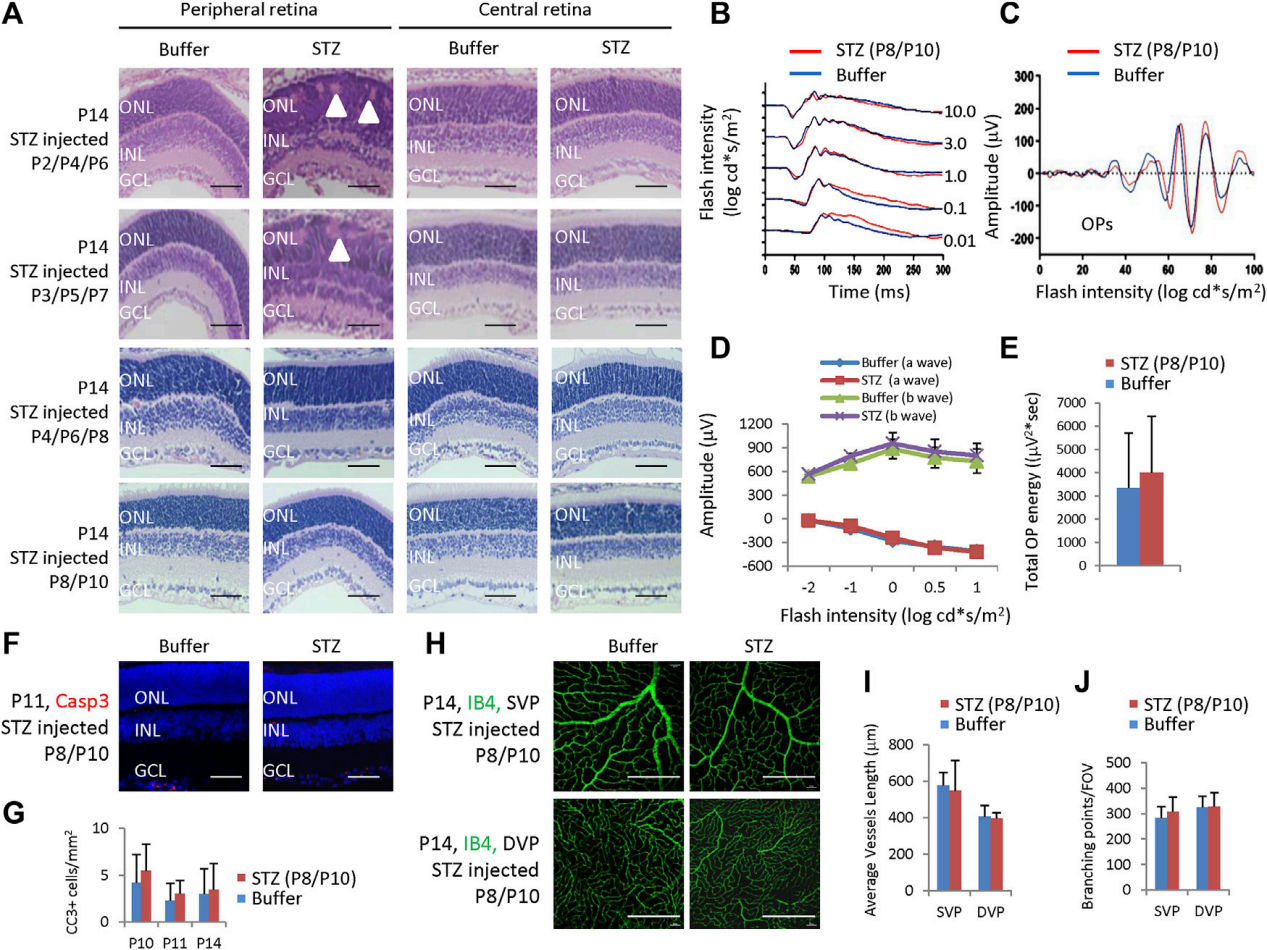
Retinal morphological changes, ERG, apoptosis, and angiogenesis in STZ-treated rats on P2/4/6, P3/5/7, P4/6/8, and P8/10. **(A)** H&E staining of P14 retinas of the control and indicated STZ-treated rats. White arrowheads indicate retinal rosettes. **(B)** ERG responses to flashes of increasing intensities from 0.01 to 10.0 cd*s/m^2^. **(C)** The OPs were extracted from dark-adapted ERGs at the intensity of 3.0 cd*s/m^2^. **(D)** Amplitude of a wave and b wave. **(E)** The total energy of the extracted OPs. **(F)** Cleaved caspase 3 staining of P11 retinas of the control and STZ (P8/P10)-treated rats. **(G)** Quantification of Cleaved caspase 3 (CC3)+ cells per mm^2^ area of the retina. **(H)** IB4 staining of P14 whole-mount retinas. **(I)** Quantification of the average vessel length of SVP/DVP of indicated groups. **(J)** Quantification of the branching points of SVP/DVP indicated groups. Error bars represent SD of measurements from six retinas of six animals (n = 6), and asterisks indicate significant differences between control and STZ-treated groups (*p < 0.05, **p < 0.01, one-way ANOVA followed by Bonferroni’s correction). Outer nuclear layer; INL, inner nuclear layer; GCL, ganglion cell layer; SVP, retinal superficial vessel plexus; DVP, retinal deep vessel plexus; FOV, field of view. Scale bar: 50 μm **(A,F)**, or 200 μm **(H)**.

We also examined rat retinas receiving STZ injection at P2/4/6, P3/5/7, P4/6/8, and P8/P10 (Figures 6C, D) and found that only retinas from the P2/4/6 and P3/5/7 groups had some rosettes in the peripheral retina; however, their central retina and all retinas from the P4/6/8 and P8/10 groups had no morphological retinal defects at P14 (Figure 8A).

Then, we focused on these rats receiving 60 mg/kg STZ at P8/P10. STZ-treated (P8/P10) rats showed normal scotopic a-wave, b-wave, and OPs at P28 (Figures 8B, C). The amplitudes of a-wave and b-waves were the same as controls (Figure 8D). The total OPs energy at the light stimulation intensity of 3.0 cd*s/m^2^ was also similar between the control and STZ-injected group (Figure 8E). The CC3+ cells were identical between the control and STZ-treated (P8/P10) groups (Figures 8F, G), and the average vessel length and branching points were also similar between these two groups (Figures 8H–J).

In summary, 60 mg/kg SC-injected STZ-induced hyperglycemia in neonatal rats only correlated with NSIR in a very short time from P1-P3. Systemic injection of 60 mg/kg STZ beyond this time window, such as on P4/6/8 or P8/10, could induce hyperglycemia, but no retinal defects, further supporting the notion that there are no significant effects of hyperglycemia on NSIR.

### 3.6 Long-term hyperglycemia has no effects on NSIR

**FIGURE 9:**
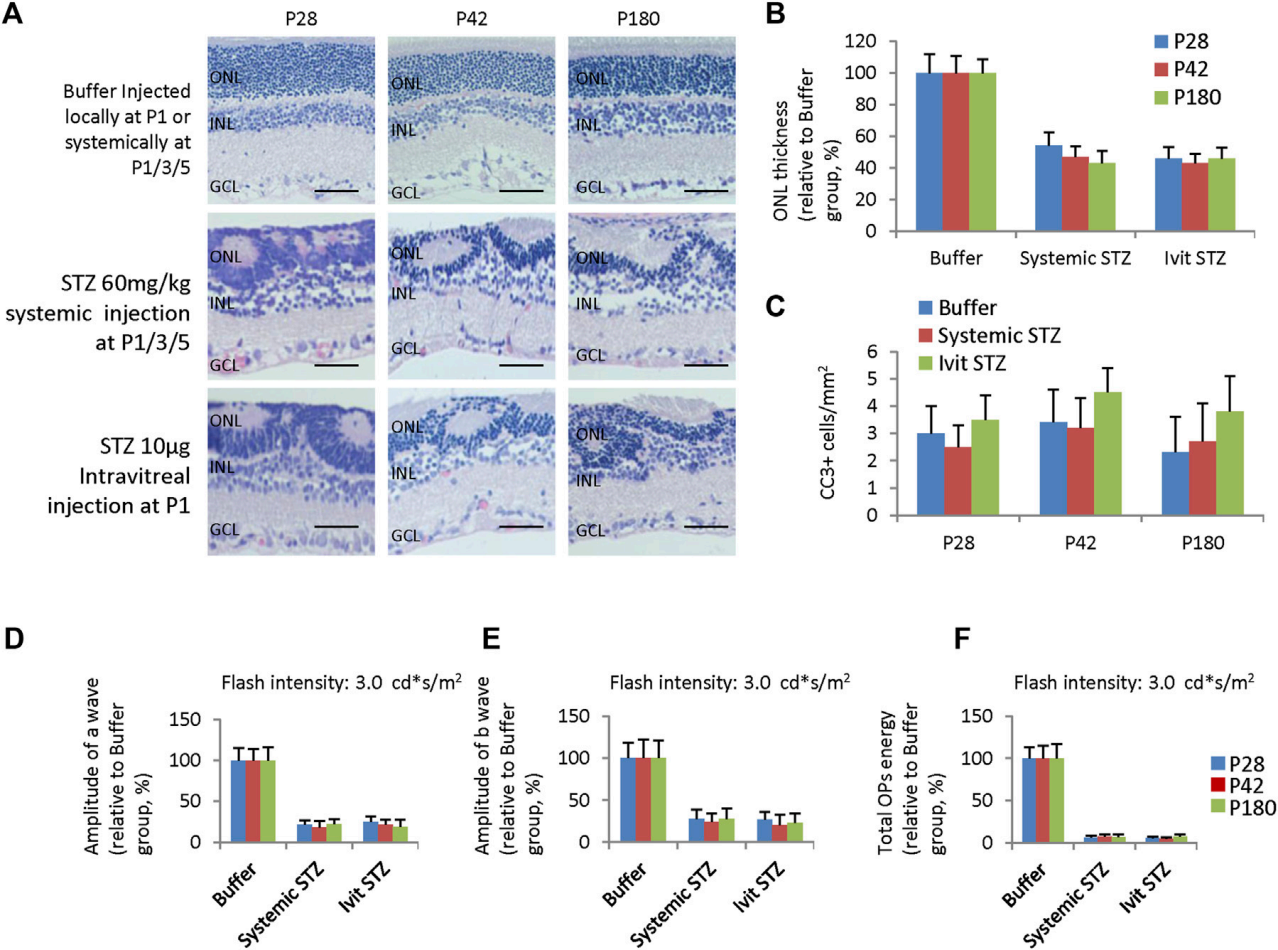
Retinal degeneration in adult rats receiving systemic STZ injection at P1/P3/P5 or intravitreal injection of STZ at P1. **(A)** H&E staining of P28, P42, and P180 retinas of the buffer and STZ-treated rats. **(B)** ONL thickness of rat retinas of indicated ages and groups relative to buffer group (%). **(C)** Quantification of Cleaved caspase 3 + (CC3) cells per mm^2^ area of the retina of suggested ages and groups. **(D)** The amplitude of a wave of ERG (Flash intensity: 3 cd*s/m^2^), relative to buffer group (%). **(E)** Amplitude of b wave of ERG (Flash intensity: 3 cd*s/m^2^), close to buffer group (%). **(F)** The total energy of the extracted OPs from dark-adapted ERGs at the intensity of 3.0 cd*s/m^2^ relative to the buffer group (%). Error bars represent the SD of measurements from six retinas of six animals (n = 6), and asterisks indicate significant differences between three time points or different treatments (*p < 0.05, **p < 0.01, one-way ANOVA followed by Bonferroni’s correction). ONL is the outer nuclear layer; INL is the inner nuclear layer; GCL is the ganglion cell layer. Ivit, Intravitreal injection. Scale bar: 50 μm.

Although NSIR is not directly related to hyperglycemia, we do not know whether long-term hyperglycemia will aggravate these pathological changes. Thus, we examined retinal morphology and function in adult rats receiving 60 mg/kg STZ SC injection at P1/P3/P5 or IVIT (10 μg) at P1. H&E staining of 4-week, 6-week, and 6-month-old rats showed similar retinal dysplasia with P14 retinas (Figures 1G, 7A, 9A), indicating little progressive retinal degeneration process. The ONL thickness was equal between these three-time points and between systemic STZ and local STZ administration, suggesting there were no significant effects of hyperglycemia on NSIR at these time points (Figure 9B).

The apoptotic peak was around P3 and P4 when STZ was delivered systemically at P1/P3 (Figure 7C) or locally at P1 (Figure 1I). CC3 staining had not identified any apoptotic events in STZ-treated retinas of P28, P42 and P180 (Figure 9C). ERG assay also showed a similar reduction of the amplitude of a-wave (Figure 9D) and b-wave (Figure 9E), and total energy of OPs (Figure 9F) between P28, P42 and P180. Thus, systemically or locally delivered STZ at P1-3 can damage the neonatal rat retina independently of hyperglycemia without progressive secondary retinal degeneration.

## 4 Discussion

In this study, we investigated the cytotoxicity of STZ on neonatal rat retinal cells. We found that, like many other chemicals such as MNU, NaIO3, and NMDA (Niwa et al., 2016; Reisenhofer et al., 2017), STZ can directly damage neonatal rat retinal cells and induce retinal degeneration (NSIR), independent of its role inducing hyperglycemia.

### 4.1 Retinal progenitor cells (RPCs) are the major target of STZ in the retina

Many chemicals, including oxidizing, methylating, and alkylating agents, as well as neurotransmitters and antibiotics, can induce retinal degeneration. Still, each has different chemical properties and special retinal target cells. For instance, NaIO3 is an oxidizing compound, specifically toxic to the RPE cells (Enzmann et al., 2006; Wang et al., 2014). MNU is a methylated nitrosourea compound with alkylating, carcinogenic, and cytotoxic properties. It interacts with DNA to yield various reaction products such as 7-methyldeoxy-guanosine adduct. Systemic administration of MNU induces retinal photoreceptor death in many animal species in adults (Tsubura et al., 2010), but also induces retinal rosettes formation and retinal dysplasia in neonatal rodents (mice and rats), suggesting toxic to retinal progenitors (Nambu et al., 1998a; Nambu et al., 1998b). NMDA is a specific agonist of the NMDA receptor mimicking the action of glutamate, the major excitatory neurotransmitter in the nervous system (Pal, 2021). Intravitreal injection of NMDA induces cell death of retinal ganglion cells in mice (Huang et al., 2014; Niwa et al., 2016).

Like MNU, STZ is an alkylating agent with carcinogenic and cytotoxic properties. Structurally, it is a moiety of MNU linked to the C-2 position of α-D-glucose (Lenzen, 2008; Zhu, 2022). Because of this glucose moiety, STZ has a high affinity for the glucose transporter Glut2, which is highly expressed in pancreatic β-cells. As such, STZ and MNU have similar chemical properties; STZ has much higher toxicity to pancreatic β-cells than MNU and is widely used to model diabetes (Lenzen, 2008; Zhu, 2022).

While MNU mainly targets photoreceptors and retinal progenitors (Tsubura et al., 2010), we found that STZ only targets retinal progenitor cells. IVIT STZ at P1 induces DNA damage, cell death, and cell cycle arrest of RPCs. Many RPCs were blocked at the G2 phase, causing reduced M-phase cells and delayed cell cycle exit, resulting in reduced late-born retinal cells. IVIT and SC injection STZ after P8, when most RPCs exit the cell cycle (Alexiades and Cepko, 1996), do not induce any measurable retinal phenotypes, supporting the notion that STZ mainly targets RPCs. Indeed, numerous studies utilized STZ in adult rats to induce diabetic retinopathy. Still, these studies seldom identified any retinal phenotypes before 2–4 weeks after STZ injection, indicating STZ has no direct toxic effects on adult rat retinal cells or post-mitotic cells (Barber et al., 1998; Asnaghi et al., 2003; Park et al., 2003; Olivares et al., 2017).

The structure and toxic action of MNU and STZ are very similar (Lenzen, 2008; Zhu, 2022), so why do MNU target photoreceptors and RPCs, but STZ only target RPCs in the retina? MNU is lipophilic, and tissue uptake through the plasma membrane is rapid; however, because of the α-D-glucose substitution, STZ is less lipophilic and must be up-taken through Glut2 (Lenzen, 2008). Thus, the possible reason is that the expression levels of Glut2 are higher in RPCs than in post-mitotic retinal cells. Therefore, RPCs can uptake STZ, but post-mitotic retinal cells cannot. It is less likely due to the blood-retinal barrier (BRB), as IVIT injection of STZ after P8 also cannot induce NSIR, which already bypasses the BRB.

### 4.2 NSIR is independent of hyperglycemia

Another interesting finding is that NSIR is not related to hyperglycemia. As STZ can damage both pancreatic β-cells and RPCs, it seems hard to exclude the effects of hyperglycemia on NSIR. We overcame this issue in two ways; first, we injected STZ (5–10 μg in total) directly into the eye by IVIT injection, thus avoiding damaging the pancreatic β-cells and hyperglycemia. The second strategy is using a low dosage of STZ (30 mg/kg) for SC injection, which induced NSIR before the appearance of hyperglycemia. Both strategies prove that STZ can induce NSIR without hyperglycemia. This is like systemic-administered MNU-induced neonatal retinopathy in mice and rats, which does not cause the death of pancreatic β-cells and hyperglycemia (Nambu et al., 1998a; Nambu et al., 1998b).

The STZ neonatal rodent models are common animal models of diabetes (Portha et al., 1989; Baig and Panchal, 2019) and are widely used to study diabetic cataract (Patil et al., 2014), diabetic nephropathy (Shinde and Goyal, 2003; Gandhi et al., 2013), diabetic neuropathy (Ashokkumar et al., 2006; Barragán-Iglesias et al., 2018), diabetes-related retinopathy of prematurity (ROP) (Kermorvant-Duchemin et al., 2013; Fu et al., 2018) and diabetic retinopathy (Mancini et al., 2013). While these studies suggested some hyperglycemia-related changes in different organs, the contributions of the direct cytotoxicity of STZ on cells in the lens, kidney, brain, and retina are generally not considered.

For instance, intraperitoneal (IP) injection of STZ on P1 rats induced transient hyperglycemia (P2-P6) but persistent NSIR features. These features were named neonatal hyperglycemia-induced retinopathy (NHIR) (Kermorvant-Duchemin et al., 2013). Similarly, daily IP injection of STZ from P1-P9 to C57BL/6 J mouse pups also induced hyperglycemia (P8-P10) and NSIR, which was named hyperglycemia-related retinopathy (HAR) (Fu et al., 2018). Both NHIR and HAR were considered novel rodent ROP models related to hyperglycemia, but not the direct cytotoxicity of STZ on retinal cells, as IVIT STZ (1 μg) in P3 rat (Kermorvant-Duchemin et al., 2013) or P1 mouse (Fu et al., 2018) did not affect weight nor blood glucose level and did not modify retinal vasculature. However, these studies only used one dose of STZ (a total of 1 μg) for IVIT. The results of IVIT STZ (1 μg) in these two studies were similar to our observation of the same STZ dosage. Still, when we increased STZ to 5–10 μg for IVIT, STZ induced direct retinal damage without hyperglycemia. Interpreting these results from STZ-induced neonatal models requires special cautions to exclude the direct cytotoxicity effects of STZ.

### 4.3 NSIR may be a valuable model for RPC DNA damage-related diseases

It is well-known that, similar to MNU, STZ can induce DNA alkylation and methylation, resulting in DNA fragmentation, which overstimulates PARP-1, diminishes the cellular NAD+ and ATP pool, and induces cell death (Yamamoto et al., 1981). The observed effects of STZ on neonatal rat retina are typical reactions of retinal progenitors to STZ-induced DNA damages, which activate the p53 pathway, thus inducing cell death and cell cycle arrest by up-regulating Fas and Cdkn1a (p21cip1).

Retinal apoptosis can further cause retinal dysplasia and delay retinal angiogenesis, as it is well known that retinal degeneration or retinal apoptosis can delay the normal development of retinal angiogenesis, as the oxygen demand in the retina is reduced (Lahdenranta et al., 2001; Pennesi et al., 2008; Zhou et al., 2018). Cell death and cell cycle arrest of RPCs reduce the pool of RPCs and delay the cell cycle exit of RPCs, resulting in fewer late-born retinal cell types. The retinal inflammation is likely a response to widespread STZ-induced retinal apoptosis (Massengill et al., 2018).

A previous study suggested that hyperglycemia-induced adiponectin (APN) deficiency, resulting in decreased photoreceptor mitochondrial metabolism and reduced Pdgfb expression, is the underlying mechanism of hyperglycemia-related retinopathy (HAR) (Fu et al., 2018). Our RNA sequencing did not identify DEGs in the APN and Pdgfb pathways. Still, the oxidative stress gene *Hmox-1* was hugely induced. It is well known that STZ can cause mitochondrial respiratory defects and oxidative stress (Raza et al., 2011; Genrikhs et al., 2017). Thus, we suggest that STZ cytotoxicity may also contribute to the previously reported HAR phenotype in neonatal mice.

Similar hyperglycemia-independent effects of STZ have been reported in skeletal muscle myoblasts (Johnston et al., 2007). *In vitro* exposures to STZ significantly impaired the proliferative capacity of myoblasts, increased reactive oxygen species (ROS), and induced G2/M phase cell-cycle arrest (Johnston et al., 2007).

As NSIR is a typical reaction of retinal progenitors to DNA damage, it may be a valuable model to study human diseases with features of RPC DNA damages, such as replication stress response (RSR)-related syndromes, including Seckel syndrome (Matos-Rodrigues et al., 2020; Matos-Rodrigues et al., 2020). NSIR is very similar to the retinal phenotypes of alpha-Cre-mediated ATRIP gene knockout mice (Matos-Rodrigues et al., 2020).

## 5 Conclusion

In conclusion, our findings reveal that NSIR in rats is not due to hyperglycemia but a direct cytotoxic effect of STZ on RPCs. Thus, NSIR may not be suitable for studying the role of hyperglycemia in the pathogenesis of retinal disease. Instead, our results suggest that NSIR is a valuable model for studying oxidative stress, DNA damage, the p53 pathway, and retinal progenitor involvement in human disease.

## Data Availability

The data presented in the study are deposited in the GEO repository, accession number GSE272311.

## Supplementary material

SUPPLEMENTARY FIGURE S1 The M phase of the cell cycle in retinal progenitors of STZ-treated rats. 10 μg STZ was intravitreal injected at P1. **(A)** Horizontal retinal sections of indicated ages and treatments were stained for nuclear (DAPI, blue) and M phase (PH3, green). **(B)** Quantification of PH3+ cells per mm^2^ area of retinal sections. Error bars represent SD of measurements from three retinas of three animals (n = 3), and asterisks indicate significant differences between control and STZ-treated groups (**P* < 0.05, ***P* < 0.01, one-way ANOVA followed by Bonferroni’s correction). ONL is the outer nuclear layer; INL is the inner nuclear layer; GCL is the ganglion cell layer; NBL is the neuroblast layer. Scale bar: 50 μm.

SUPPLEMENTARY FIGURE S2 Retinal apoptosis (TUNEL) in STZ-treated rats (P1/P3/P5). **(A)** TUNEL of P2-P8 retinas of the control and STZ-treated rats. **(B)** Quantification of TUNEL+ cells per mm^2^ area of retinal sections. Error bars represent SD of measurements from six retinas of six animals (n = 6), and asterisks indicate significant differences between control and STZ-treated groups (**P* < 0.05, ***P* < 0.01, one-way ANOVA followed by Bonferroni’s correction). ONL is the outer nuclear layer; INL is the inner nuclear layer; GCL is the ganglion cell layer; NBL is the neuroblast layer. Scale bar: 50 μm.

SUPPLEMENTARY FIGURE S3 The S phase of the cell cycle in retinal progenitors of STZ-treated rats (P1/P3/P5). STZ (60 mg/kg body weight) was subcutaneously injected at P1, P3, and P5. EdU (30 μg/g body weight) was subcutaneously injected 30 min before retina harvesting. **(A)** Horizontal retinal sections of indicated ages and treatments were stained for nuclear (DAPI, blue) and labeled by an EdU click-it kit (purple). **(B)** Quantification of EdU + cells per mm^2^ area of retinal sections. **(C)** Quantification of EdU + cells per mm^2^ area of retinal sections of P6/P8 retinas. As the P6/P8 retina had very few dividing cells, the peripheral retina was divided into four areas according to its distance from the iris root. Error bars represent SD of measurements from six retinas of six animals (n = 6), and asterisks indicate significant differences between control and STZ-treated groups (**P* < 0.05, ***P* < 0.01, one-way ANOVA followed by Bonferroni’s correction). ONL is the outer nuclear layer; INL is the inner nuclear layer; GCL is the ganglion cell layer; NBL is the neuroblast layer. Scale bar: 50 μm.

SUPPLEMENTARY FIGURE S4 The effects of STZ on retinal proliferation at P6. STZ (60 mg/kg body weight) was subcutaneously injected at P1, P3, and P5. EdU (30 μg/g body weight) was subcutaneously injected 30 min before retina harvesting. **(A)** P6 horizontal retinal sections of the BUF group and STZ treatment group were stained for the pan-cell cycle (Ki67, green), S-phase (EdU, red), and nuclei (DAPI, blue). The location of the optic nerve is marked. Scale bar = 1000 μm. **(B)** Schematic diagram based on the results in Figure A, showing that EdU + or Ki67+ progenitor cells in the BUF group were concentrated in the peripheral retina at P6, and all progenitors in the central retina had all exited the cell cycle and began to differentiate; the EdU + or Ki67+ progenitor cells in the STZ treatment group were widely distributed throughout the whole retina at P6, and many progenitors in the central retina had not exited the cell cycle and did not begin to differentiate.

SUPPLEMENTARY FIGURE S5 The effects of STZ on retinal proliferation at P8. STZ (60 mg/kg body weight) was subcutaneously injected at P1, P3, and P5. EdU (30 μg/g body weight) was subcutaneously injected 30 min before retina harvesting. **(A)** P8 horizontal retinal sections of the BUF group and STZ treatment group were stained for the pan-cell cycle (Ki67, green), S-phase (EdU, red), and nuclei (DAPI, blue). The location of the optic nerve is marked. Scale bar:1000 μm. **(B)** Schematic diagram based on the results in Figure A, showing that EdU + or Ki67+ progenitor cells in the BUF group were concentrated in the most peripheral retina at P8, and all progenitors in most parts of the retina had all exited the cell cycle and began to differentiate; the EdU + or Ki67+ progenitor cells in the STZ treatment group were distributed in the mid-peripheral retina at P8, these cells had not exited the cell cycle and did not begin to differentiate.
