# Supplementary figures and images for: Hyperglycemia-independent neonatal streptozotocin-induced retinopathy (NSIR) in rats
Source: Front Pharmacol. 2024 Jul 23;15:1395887. doi: 10.3389/fphar.2024.1395887 (PMC11300211; doi:10.3389/fphar.2024.1395887)

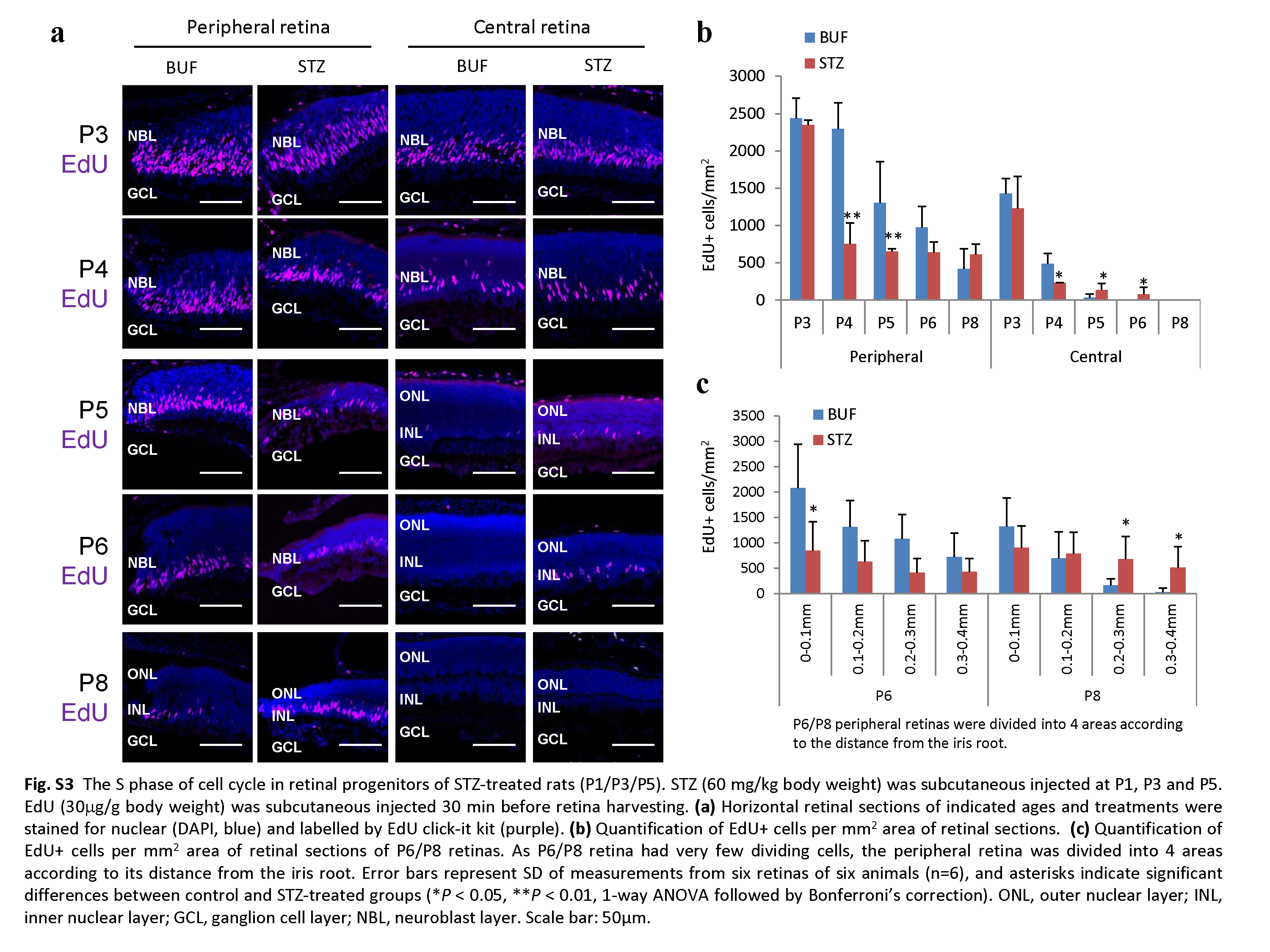

Supplement: Supplementary file 1 [file Image3.TIFF]

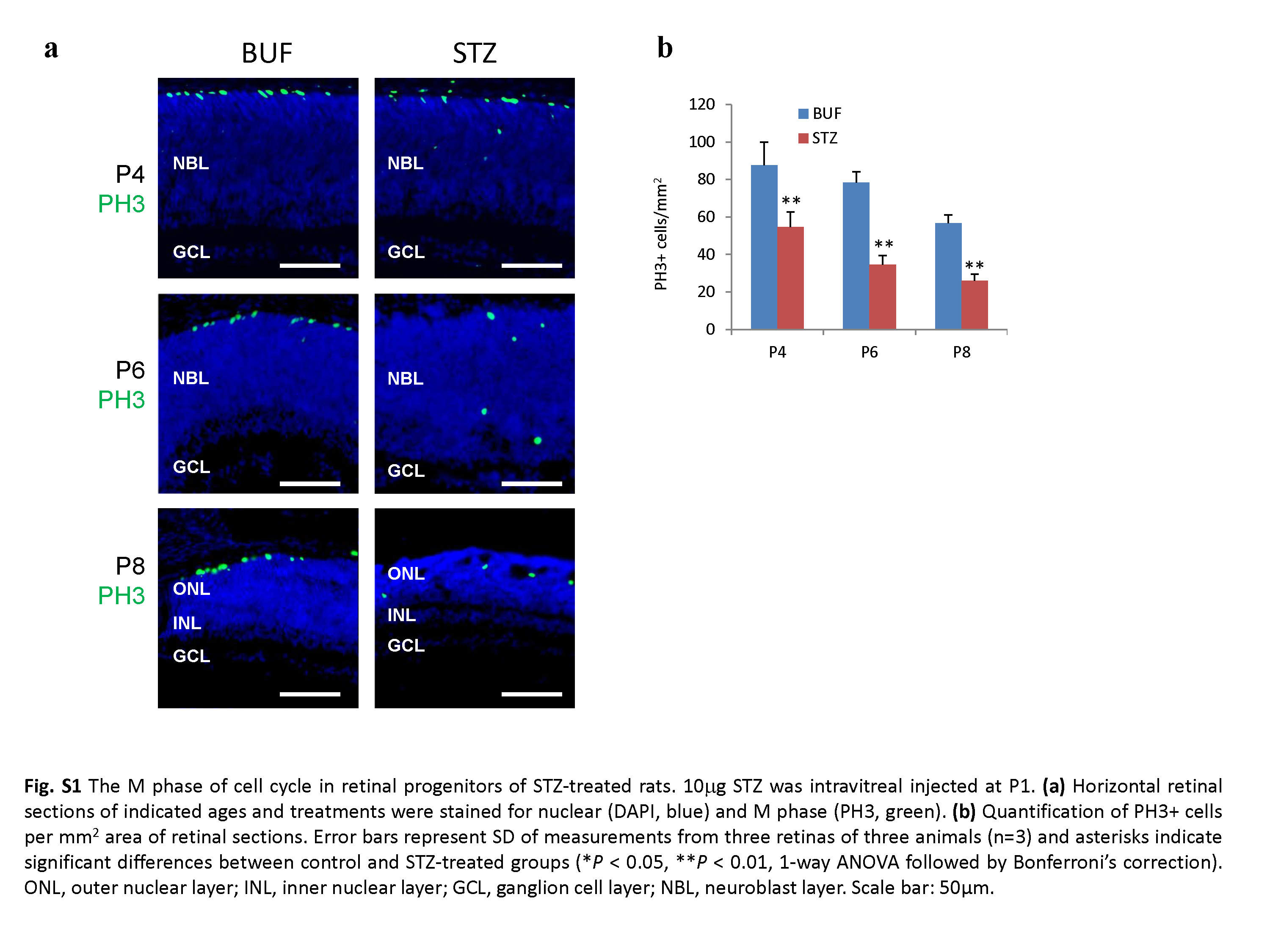

Supplement: Supplementary file 3 [file Image1.TIFF]

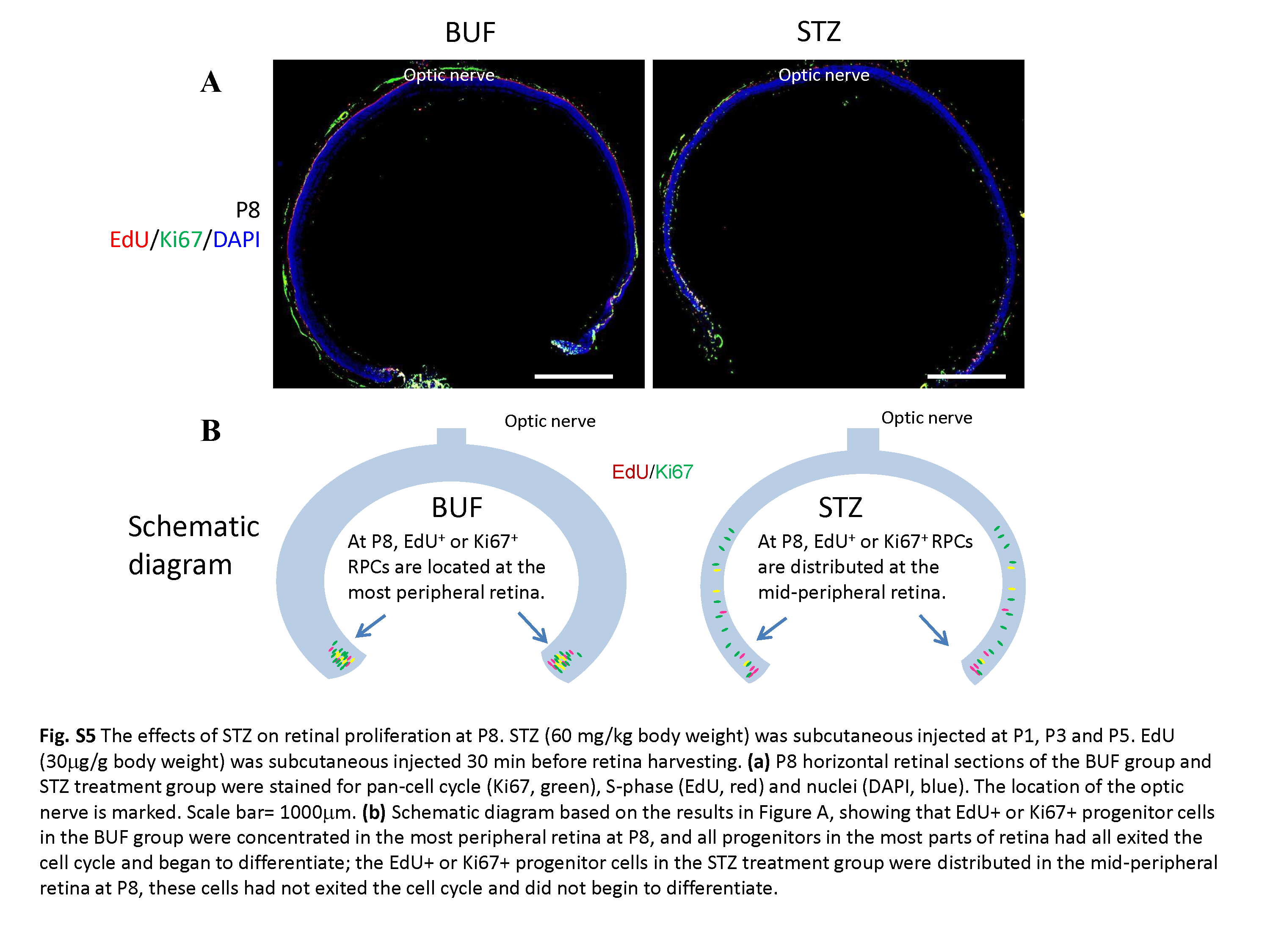

Supplement: Supplementary file 4 [file Image5.TIFF]

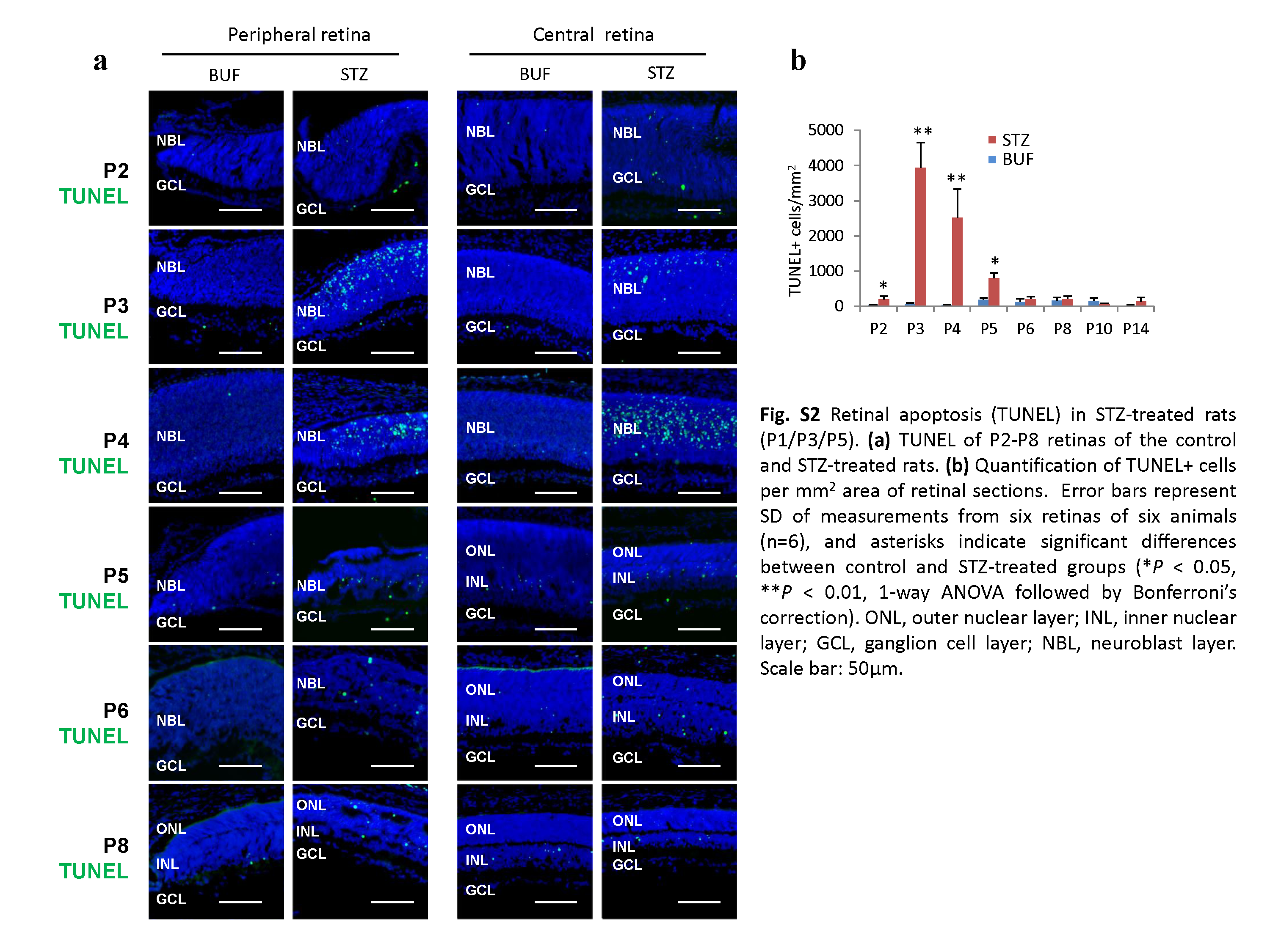

Supplement: Supplementary file 6 [file Image2.TIFF]

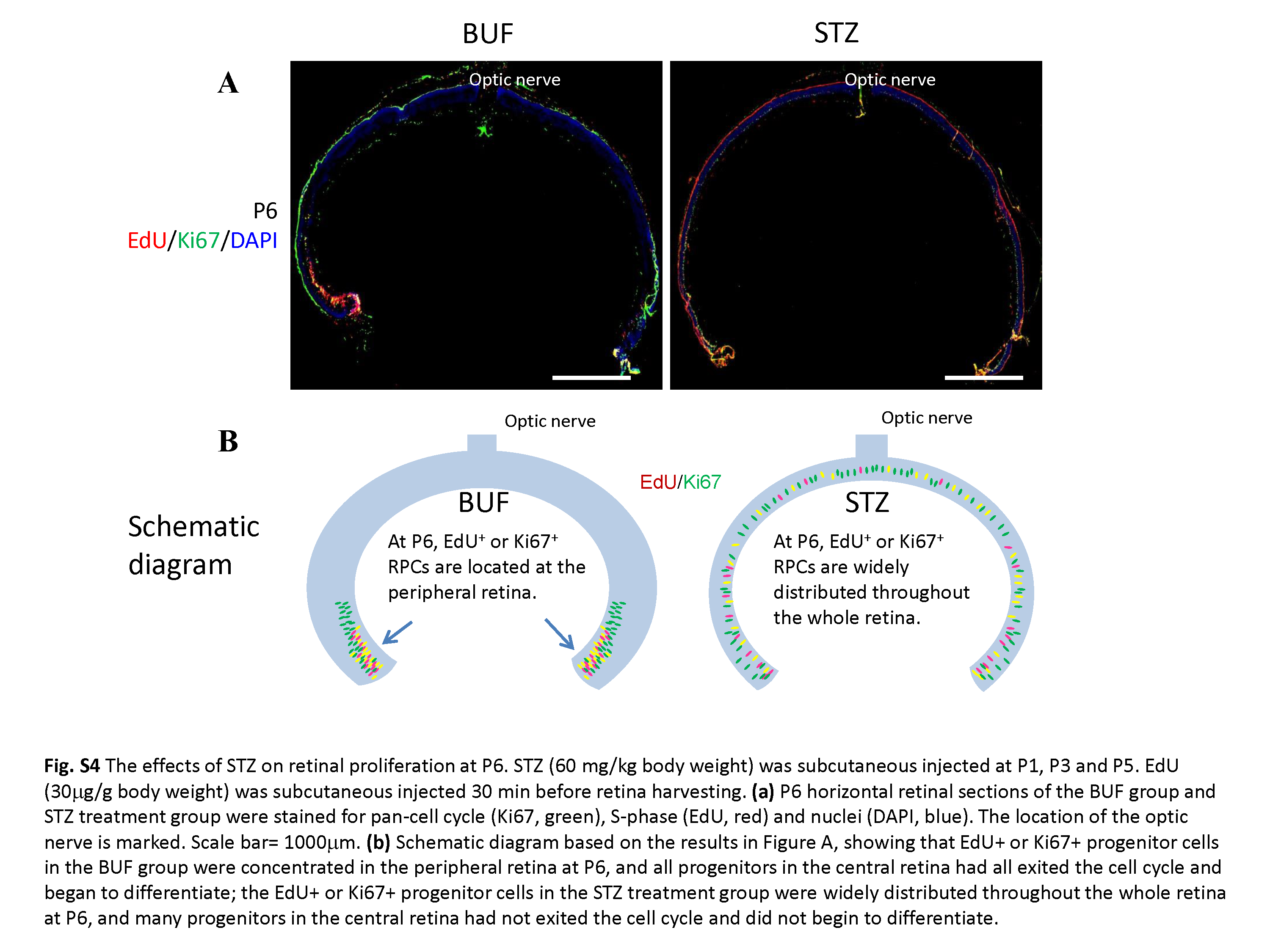

Supplement: Supplementary file 7 [file Image4.TIFF]
